# Novel chemical entities inhibiting *Mycobacterium tuberculosis* growth identified by phenotypic high-throughput screening

**DOI:** 10.1038/s41598-022-19192-7

**Published:** 2022-09-01

**Authors:** Anuradha Kumar, Somsundaram Chettiar, Brian S. Brown, Julie Early, Juliane Ollinger, Megan Files, Mai A. Bailey, Aaron Korkegian, Devon Dennison, Matthew McNeil, James Metz, Augustine Osuma, Michael Curtin, Aaron Kunzer, Gail Freiberg, Milan Bruncko, Dale Kempf, Tanya Parish

**Affiliations:** 1 Infectious Disease Research Institute, Seattle, WA 98102 USA; 2 AbbVie Inc., North Chicago, IL USA; 3 Center for Global Infectious Disease Research, Seattle Children’s Research Institute, Seattle, WA 98109 USA

**Keywords:** Medicinal chemistry, Drug screening, Phenotypic screening, Tuberculosis

## Abstract

We performed a high-throughput phenotypic whole cell screen of *Mycobacterium tuberculosis* against a diverse chemical library of approximately 100,000 compounds from the AbbVie corporate collection and identified 24 chemotypes with anti-tubercular activity. We selected two series for further exploration and conducted structure–activity relationship studies with new analogs for the 4-phenyl piperidines (4PP) and phenylcyclobutane carboxamides (PCB). Strains with mutations in MmpL3 demonstrated resistance to both compound series. We isolated resistant mutants for the two series and found mutations in MmpL3. These data suggest that MmpL3 is the target, or mechanism of resistance for both series.

## Introduction

Despite being widely recognized as a global health priority, tuberculosis (TB) remains a leading cause of death globally, and the deadliest bacterial infectious disease^1^, with 1.5 million deaths in 2020^2^. There were half a million cases of multi-drug resistant TB (MDR-TB), and an increased prevalence of strains resistant to second-line and reserve medicines (XDR-TB)^2^. MDR-TB is the largest contributor to antimicrobial resistance (AMR) and is predicted to cause a quarter of the 10 million deaths from AMR infections by 2050. Although there has been a slow decline in TB cases, the impact of the Covid-19 pandemic is likely to exacerbate the problem and reverse progress. Therefore, new drugs that can treat drug-resistant TB are urgently needed. The pipeline of compounds in development remains small given the high attrition rate at all stages of discovery and development. Therefore, a much larger set of antitubercular agents are needed in order to guarantee an adequate number of new clinical candidates.

The identification of new molecules which target *Mycobacterium tuberculosis*, the causative agent of TB, has been the subject of numerous screening campaigns^3–5^. Phenotypic screening with large commercial libraries has been successful in identifying agents with antimycobacterial activity. In this study we performed a high-throughput screen against a diverse chemical library of approximately 100,000 compounds from the AbbVie corporate collection. Here we disclose the chemotypes of hits from this screen and describe initial efforts to progress these compounds for TB drug discovery.

## Results

### Identification of anti-tubercular compounds from the AbbVie diversity library

**Figure 1:**
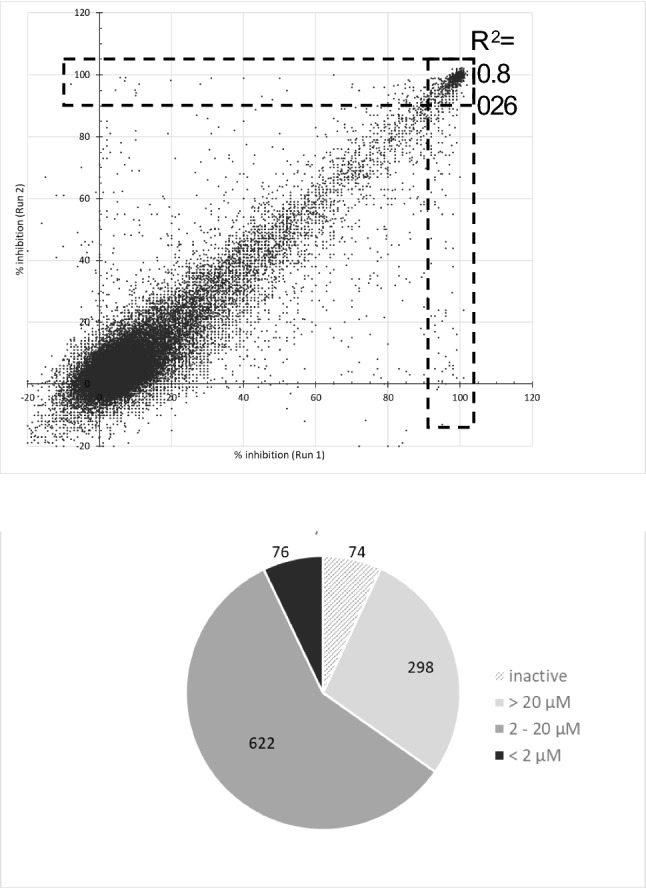
A high throughput screen for inhibitors of *M. tuberculosis* growth. (**A**) We tested a library of 98,347 compounds for activity. *M. tuberculosis* was grown in the presence of 20 µM test compound for 5 days in 384-well plates in duplicate. Growth inhibition was calculated relative to the control (DMSO). R^2^ using Pearson’s correlation coefficient is shown. Compounds with > 90% inhibition are boxed with a dashed-line. (**B**) Hits were reconfirmed in a dose response assay. 1070 hits were tested and the IC_90_ against *M. tuberculosis* in liquid culture determined. The number of compounds in each category of potency is indicated.

Our overall goal was to identify new chemotypes for development as anti-tubercular agents. We had previously developed a high throughput assay which could be adapted to 384-well format allowing the screening of large compound collections^6–8^. We selected a set of ~ 100,000 molecules from the AbbVie corporate collection. This small molecule collection contained a diversity of pharmacophores and chemical scaffolds representing the larger AbbVie collection. We screened 98,347 compounds in duplicate against wild-type *M. tuberculosis* H37Rv at a fixed concentration of 20 µM. Growth was measured after 5 days and % growth inhibition calculated. The two runs showed good agreement with an R^2^ value of 0.80; we identified 1311 compounds which inhibited growth of *M. tuberculosis* by > 90% in at least one of the runs (Fig. 1A). The hit rate of 1.3% was similar to that seen in our previous screens.

We confirmed the activity of 1070 hits from the primary screen using fresh compound supply (Fig. 1B). Compounds were tested as 10-point serial dilutions and we determined the MIC for each compound, defined as the compound concentration at which 90% of growth was inhibited^9^. The reconfirmation rate was high, with 93% of compounds showing some inhibition of growth (> 30%) at 20 µM. Approximately two thirds of the hits had MIC < 20 µM, and another 28% were active, but with MIC > 20 µM (Fig. 1B). Of interest, 76 of the confirmed hits were potent, with MIC < 2 µM, representing about 0.1% of the library.

**Figure 2:**
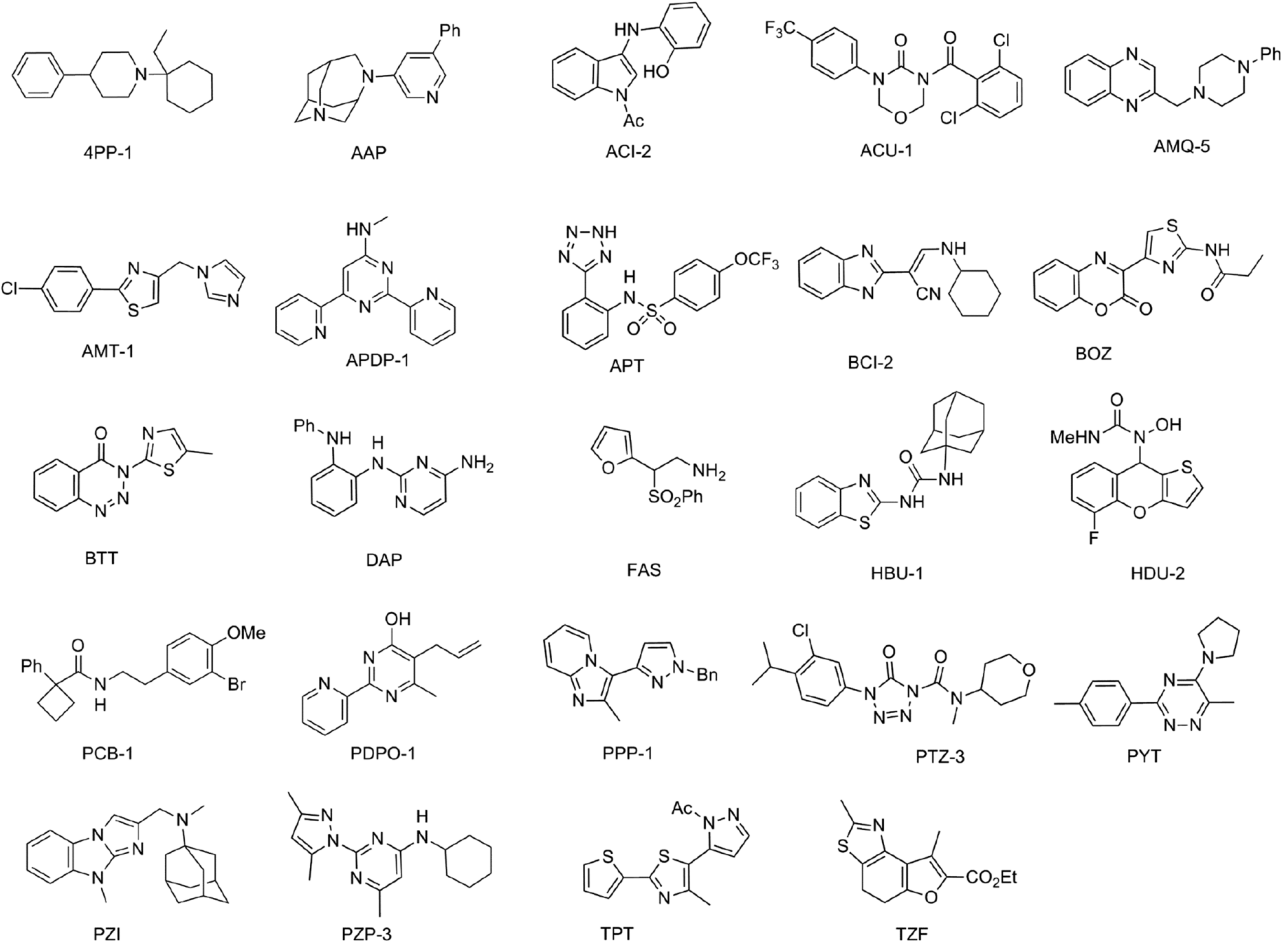
Structures of Novel Chemotypes. 24 distinct chemotypes were confirmed as hits from the primary screen. The structure of a representative from each chemotype is shown. Abbreviations: Ph = phenyl, Ac = acetyl, Me = methyl, Bn = benzyl, Et = ethyl.

We analyzed the confirmed hits and identified a number of distinct chemotypes (Fig. 2); some of these were represented by multiple analogs in the screen, while others were singletons. We determined cytotoxicity against HepG2 cells (IC_20_) and found a range of activity with some compounds showing low cytotoxicity (IC_20_ > 40 µM) or a high selectivity index (SI: HepG2 IC_20_/Mtb MIC) (Table 1).

**Table 1:** Activity of novel chemotypes against *M. tuberculosis*.

Chemotype	Molecule	% Inhibition^1^		MTB^2^	HepG2^3^
		Run 1	Run 2	MIC (µM)	IC_20_ (µM)
4-phenylpiperidine	4PP-1	100	99	6.3	> 80
Aminoarylpyridine	AAP	99	97	23	27
Acetyl indole	ACI-2	100	100	4.5	30
Acyl cyclic urea	ACU-1	98	98	7.6	65
Aminomethylquinoxaline	AMQ-5	98	99	7.8	2.0
Aminomethylthiazole	AMT-1	98	96	35	34
Aminopyridylpyrimidine	APDP-1	99	100	10	0.028
Aminophenyltetrazoles	APT	99	99	6.9	0.47
Benzimidazolyl cyanoimines	BCI-2	100	100	9.6	0.85
Benzoxazinones	BOZ	99	100	4.8	37
Benzotriazinylthiazoles	BTT	100	100	4.8	37
Diaminopyrimidines	DAP	99	99	0.7	0.83
Furanylaminosulfones	FAS	100	100	9.5	2.0
Hydrophobic urea	HBU-1	100	100	4.2	8.2
Hydroxyureas	HDU-2	99	100	19	42
Phenylcyclobutanecarboxamides	PCB-1	100	100	6.9	33
Pyrazolylpyrrolopyridines	PDPO-1	99	96	23	2.8
Pyridylpyrimidines	PPP-1	100	98	38	4.3
Phenyltetrazolones	PTZ-3	96	6	5.2	26
Pyrrolotriazines	PYT	100	98	11	23
Pyrazoloindoles	PZI	98	98	9.3	15
Pyrazolylpyrimidines	PZP-3	98	98	4.5	47
Thienylpyrazolylthiazoles	TPT	99	99	7.1	> 80
Thiazolyl Furans	TZF	99	99	18	> 80

We determined the MIC of a representative molecule for each chemotype.

^1^% Inhibition of *M. tuberculosis* growth after 5 days in the primary screen (duplicated runs).

^2^MIC is the concentration required to achieve 90% inhibitions of growth of *M. tuberculosis* in aerobic culture (n ≥ 2).

^3^IC_20_ is the concentration required to achieve 20% inhibition of HepG2 cells (n = 2).

We evaluated each chemotype according to both biological activity and chemical features. Of the 24 chemotypes, six had a lack of in vitro selectivity for *M. tuberculosis* over eukaryotic cells. Of the remaining, two series with attractive chemical properties were selected for further investigation; the 4-phenyl piperidines (4PP), which had three confirmed actives from the screen with MIC ranging from 6.3 to 23 µM and a phenylcyclobutane carboxamide (PCB), which was a singleton with an MIC of 6.9 µM. We considered these two seriest o be the highest priority series based on a combination of their physicochemical properties, low cytotoxicity, lack of structural alerts, potency in the screen, and the possibility to conduct structure activity relationship studies.

### Identification of compounds with common mechanism of action or resistance

Phenotypic screening has identified a small number of vulnerable proteins which are targeted by multiple chemical scaffolds. Three of the most common are the membrane proteins, MmpL3, QcrB and DprE1, which are frequently linked to the mechanism of action of novel chemical entities in development. We wanted to determine if the chemotypes we identified might target these pathways. We selected representative compounds from each of the 24 chemotypes and tested them for activity against *M. tuberculosis* strains carrying mutations conferring resistance to other compound classes; we selected strains carrying either MmpL3_F255L_, QcrB_A396T_ or DprE1_C387S_^9–12^. Lower activity against one of these strains would suggest that the protein is either the direct target or is involved in its mechanism of action.

We determined the MIC for each chemotype against the mutant strains (Table 2). Four chemical series had at least one molecule with ≥ threefold lower activity against the MmpL3 mutant strain (Table 2). Two chemotypes (PYT and TPT) showed ≥ threefold lower activity against the QcrB mutant strain, suggesting that the mechanism of action involves disruption of the electron transport chain. None of the compounds showed lowered potency against the DprE1 mutant strain. Based on these data we prioritized the two series that appear to target MmpL3 and deprioritized the two series that appear to target QcrB; the latter was deprioritized due to a concern over the redundancy and potential flexibility in the respiratory chain of *M. tuberculosis*.

**Table 2:** Identification of common targets or mechanisms of resistance.

Chemotype	Molecule	Wild-type	MmpL3 F255L	Fold change	Structure
		IC_90_ (µM)	IC_90_ (µM)		
4-phenylpiperidine	4PP-1	6.3	68	**11**	See Fig. 2
4-phenylpiperidine	4PP-31	2.7	45	**15**	See Table 3
4-phenylpiperidine	4PP-44	5.2	18	**3.5**	See Table 3
Aminopyridylpyrimidine	APDP-1	10	9.2	0.9	See Fig. 2
Aminopyridylpyrimidine	APDP-2	33	15	0.5	See Fig. 3
Aminopyridylpyrimidine	APDP-3	6.3	22	**3.5**	See Fig. 3
Hydrophobic urea	HBU-1	4.2	6	1.4	See Fig. 2
Hydrophobic urea	HBU-2	4.9	39	**8.0**	See Fig. 3
Hydrophobic urea	HBU-3	29	71.0	2.4	See Fig. 3
Hydrophobic urea	HBU-4	12	> 100	**> 8.3**	See Fig. 3
Hydrophobic urea	HBU-5	13	6.9	0.5	See Fig. 3
Pyrazolylpyrimidines	PZP-1	5.2	> 100	**> 19**	See Fig. 3
Pyrazolylpyrimidines	PZP-2	7.4	> 100	**> 13**	See Fig. 3
Pyrazolylpyrimidines	PZP-3	4.5	46	**10**	See Fig. 2

Chemotype	Molecule	Wild-type	QcrB A396T	Fold-change	Structure
		IC_90_ (µM)	IC_90_ (µM)		
Pyrrolotriazines	PYT	11	62	**5.8**	See Fig. 2
Thienylpyrazolylthiazoles	TPT	7.1	63	**8.9**	See Fig. 2

**Figure 3:**
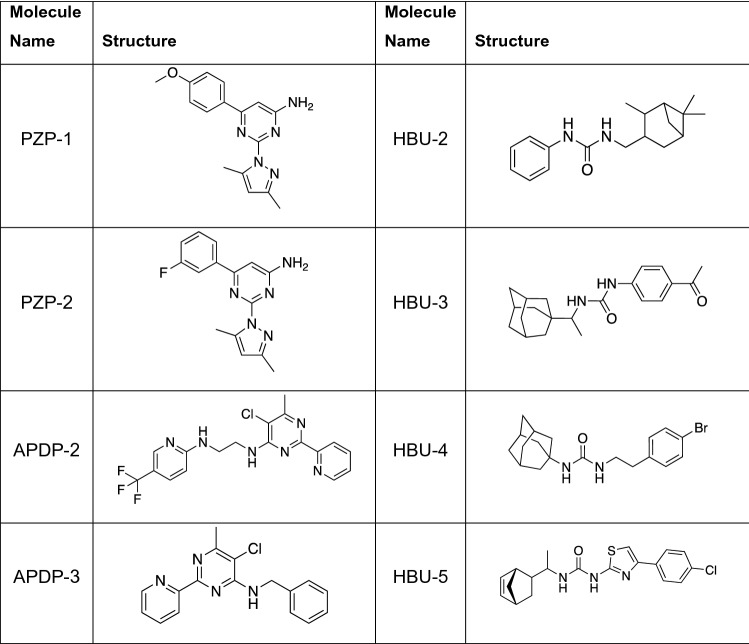
Structures of molecules tested for activity against *M. tuberculosis* strains.

We determined the MIC against wild-type *M. tuberculosis* and strains with mutations in either MmpL3 or QcrB. The fold change with respect to the wild-type MIC; in bold if change > three-fold. Structures are shown in Figs. 2 and 3.

**Table 3:** Structure–activity relationship for 4PP chemotype.

Molecule	Structure	MIC (µM)	clogP	TPSA (Å^2^)
4PP-1		6.3 ± 2.7	5.06	3.24
4PP-2		2.0 ± 0.5	6.61	3.24
4PP-3		6.8 ± 0.9	6.12	3.24
4PP-4		21 ± 9.0	4.58	3.24
4PP-5		> 20	4.83	37.38
4PP-6		> 20	5.35	20.31
4PP-7		> 20	4.18	32.34
4PP-8		> 20	4.2	23.55
4PP-9		> 20	3.29	37.38
4PP-10		> 20	3.8	20.31
4PP-11		> 20	2.63	32.34
4PP-12		> 20	2.65	23.55
4PP-13		> 20	3.38	32.34
4PP-14		> 20	3.42	20.31
4PP-15		> 20	− 0.74	40.54
4PP-16		2.7 ± 0.8	5.81	3.24
4PP-17		> 20	5.13	6.48
4PP-18		> 20	5.17	29.54
4PP-19		> 20	2.3	40.54
4PP-20		> 20	4.63	20.31
4PP-21		> 20	3.58	6.48
4PP-22		> 20	3.63	29.54
4PP-23		> 20	2.87	23.47
4PP-24		> 20	3.97	12.47
4PP-25		> 20	4.03	6.48
4PP-26		> 20	2.42	12.47
4PP-27		> 20	2.49	6.48
4PP-28		2.8 ± 0.7	5.34	3.24
4PP-29		4.1 ± 0.0	5.18	3.24
4PP-30		> 20	4.43	27.03
4PP-31		2.7 ± 2.4	5.57	3.24
4PP-32		> 20	3.36	16.13
4PP-33		7.5 ± 4.0	3.88	21.7
4PP-34		23 ± 1.4	3.47	21.7
4PP-35		6.3 ± 0.1	4.65	3.24
4PP-36		> 20	4.4	20.31
4PP-37		> 20	4.13	27.03
4PP-38		> 20	3.31	32.34
4PP-39		> 20	4.21	29.54
4PP-40		> 20	3.59	23.47
4PP-41		> 20	4.19	3.24
4PP-42		> 20	3.81	21.7
4PP-43		5.2 ± 1.9	6.61	3.24
4PP-44		5.2 ± 5.0	5.07	3.24
4PP-45		> 20	4.21	31.92
4PP-46		> 20	4.32	31.92
4PP-47		9.6 ± 2.2	5.06	19.03
4PP-48		1.6 ± 0.0	5.17	19.03
4PP-49		> 5	6.08	6.48
4PP-50		> 10	4.54	6.48
4PP-51		> 20	4.47	3.24
4PP-52		> 20	5.07	3.24
4PP-53		> 20	4.31	12.47

MICs were determined against wild-type *M. tuberculosis* after 5 days growth (n ≥ 2). cLogP and TPSA (total polar surface area) were generated by Collaborative Drug Discovery (CDD).

### Structure–activity-relationship of the 4PP series

We selected the 4PP series for further work as it had promising in vitro activity, some selectivity, and appears to target MmpL3, a high value drug target due to its essentiality and vulnerability in vitro and in vivo^13^. The three hits from the primary screen had poor physicochemical properties, with high cLogP values, so our focus was on addressing this liability while maintaining anti-bacterial activity. We conducted a structure activity relationship (SAR) study which included identifying substitutions that would improve the physicochemical properties.

We obtained or synthesized a set of ~ 50 analogs and tested them for activity against *M. tuberculosis* (Table 3). Our focus was to determine whether we could reduce cLogP and retain potency. Analogs were designed to explore the chemical space and to determine which parts of the molecules were amenable to substitution with a view to then generating molecules with improved physicochemical properties. First, we evaluated analogs of the initial hit (**4PP-1**, MIC = 6.3 µM) with a hydrogen at the 4-position and a 1-ethylcyclohexyl substitution at the N-1 position of the piperidinyl moiety. **4PP-2** with a 4-(p-tert-butylphenyl) group at the 4-position had improved activity with an MIC of 2.0 µM, whereas **4PP-3** with a 4-(p-tert-butylphenyl) group at the 4-position and a cyclohexylmethylene group at the N-1 position of the piperidinyl moiety was similar in activity to the seed hit **4PP-1** (MIC = 6.8 µM compared to 6.3 µM). Its close analog, **4PP-4**, with a phenyl group at the 4-position had modest activity (21 µM). Replacing the methylene spacer and linking the cyclohexyl group to the N-1 position via a sulfonamide (**4PP-5**) or an amide (**4PP-6**) group improved the cLogP and the total polar surface area (TPSA), but molecules lost whole-cell activity. Replacing the cyclohexylmethylene group with a urea-linked tert-butyl (**4PP-7**) or piperidine (**4PP-8**) also resulted in a loss of activity. A similar trend was observed with the corresponding 4-phenyl substituted analogs **4PP-9, 4PP-10, 4PP-11** and **4PP-12** which were designed to reduce cLogP (but lost activity). Furthermore, three additional 4-phenyl substituted moieties did not furnish activity; **4PP-13** with a N-1 urea-linked cyclohexyl, and **4PP-14** with a N-1 amide linked tert-butyl, and **4PP-15** with a N-1 acetic acid substitution.

In contrast, removing the methylene spacer between the *N*-piperidinyl and cyclohexyl group, as in **4PP-16** maintained activity (MIC = 2.7 µM). Addition of a polar 4-*N,N*-dimethylamino group off the N-1 cyclohexyl ring (**4PP-17**) was detrimental to the activity. Similarly, addition at this position of an ester (**4PP-18**), a carboxylic acid (**4PP-19**), or substitution with a cyclohexanone group (**4PP-20**) showed a loss in activity. We saw a similar loss of activity in the corresponding 4-phenyl substituted analogs containing a dimethylamino, ester, or hydroxyl addition to the cyclohexyl ring (**4PP-21**, **4PP-22**, and **4PP-23**). Replacing the cyclohexyl ring with a tetrahydropyra-4-yl (**4PP-24**) or *N*-methylpiperidin-4-yl (**4PP-25**) also yielded inactive analogs with MICs > 20 µM. As before, the corresponding molecules (**4PP-26** and **4PP-27**) with the 4-phenyl group (with reduced cLogP) were also inactive (MIC > 20 µM). Thus, our initial attempts to reduce the clogP while retaining activity were unsuccessful.

We also explored a variety of modifications and substitutions of the phenyl group at the 4-position of the piperidinyl core, retaining the cyclohexylmethylene at the N-1 position. Molecules from the original hit expansion with m-chloro and m-bromo substitutions on the phenyl group (**4PP-28** and **4PP-29**) were active with MICs of 2.8 µM and 4.1 µM, respectively. A related analog with a p-cyano group was inactive (**4PP-30**). Two additional analogs were synthesized replacing the phenyl group; **4PP-31** with a 1-naphthyl group, was active with an MIC of 2.7 µM, but **4PP-32** with a 4-pyridinyl was inactive.

The other two primary hits had spiro-substitutions at the 4-position of the piperidinyl moiety. **4PP-33** which had a spiroindene was active with an MIC of 7.5 µM and contained a benzo[d][1,3]dioxol-5-ylmethyl at the N-1 position of the piperidinyl moiety. **4PP-34**, containing an oxygen heterocycle in place of the spiroindene ring was less active with an MIC of 23 µM. Replacement at the N-1 position with a simple cyclohexyl ring (**4PP-35**) resulted in an active molecule with an MIC of 6.3 µM. However, five separate analogs with 4,4-disubstitution on the piperidinyl core were evaluated and all found to be inactive (**4PP-36**–**4PP-40**).

We further explored replacing the N-1 cyclohexyl group with aromatic or branched aliphatic groups. In the case of aromatic substitutions, both **4PP-41** with a benzyl group, and **4PP-42** with a benzo[d][1,3]dioxol-5-ylmethyl group were inactive. On the other hand, analogs containing the aliphatic 2,4,4-trimethylpentan-2-yl substituted piperidinyl (**4PP-43** and **4PP-44**) had an MIC of 5.9 µM and 5.2 µM, respectively. Varying the substituent on the 4-position of the piperidinyl while retaining the branched chain aliphatic group at the N-1 position had mixed results. Analogs containing a 1*H*-pyrrolo[2,3-b]pyridin-3-yl group were inactive (**4PP-45** and **4PP-46**), but those containing a 1*H*-indol-3-yl retained activity (**4PP-47** and **4PP-48**, MIC of 9.6 and 1.6 µM respectively).

Finally, replacement of the central piperidinyl ring demonstrated that few modifications were tolerated. We observed a loss in activity when the piperidinyl core was replaced with a piperazinyl core as demonstrated by compounds **4PP-49** and **4PP-50** (compared with **4PP-43** and **4PP-44**). The addition of a small 3-fluoro group on the piperidinyl core was also not tolerated as in the case of **4PP-51**, **4PP-52**, and **4PP-53**.

### Structure–activity-relationship of the PCB series

We selected a second series from the primary screen for follow-up. The PCB chemotype was poorly represented in the screening library, with only one confirmed active with an MIC of 6.9 µM (**PCB-1**). However, upon hit expansion from within the AbbVie chemical database a close analog (**PCB-2**) provided an excellent starting point for this series, with good activity (MIC of 3.4 µM), and reasonable in vitro human unbound microsomal clearance (Cl_int,u_ 35.6 L/hr/kg). However, the compound showed poor exposure in mouse PK experiments (AUC 58 ng hr/mL @ 30 mg/kg, po). Despite having a reasonable PAMPA permeability (87 × 10^−6^ cm/s) value, its low critical aggregation concentration (3.3 µM) indicated dissolution related problems, likely due to its high lipophilicity. Early hit expansion generally identified compounds with significantly higher clearances, so our studies focused on finding compounds with better physical properties and microsomal stability, in addition to improved activity (Table 4).

**Table 4:** Structure–activity relationship for PCB chemotype.

Molecule	Structure	MIC^1^ (µM)	human Cl_int_^2^ (L/hr/kg)	clogP	TPSA (Å^2^)
PCB-1		6.9 ± 1.7	79.7	4.73	38.3
PCB-2		3.4 ± 0.6	25.6	5.35	49.3
PCB-3		> 20	12.5	3.66	58.6
PCB-4		12 ± 2.6	52.1	3.74	47.6
PCB-5		4.9 ± 1.7	236	4.89	29.1
PCB-6		5.4 ± 0.3	35.6	4.89	29.1
PCB-7		3.7 ± 2.7	270	5	29.1
PCB-8		> 20	69.7	3.14	41.5
PCB-9		6.9 ± 2.9	273	5.43	29.1
PCB-10		9.3 ± 1.0	14.4	5.02	54.9
PCB-11		> 20	9.66	2.75	67.8
PCB-12		7.3 ± 0.3	47.3	4.11	46.2
PCB-13		> 20	266	5.29	34.0
PCB-14		> 20	447	5.11	20.3
PCB-15		9.7 ± 6.7	559	5.49	29.1
PCB-16		5.0 ± 1.6	552	5.49	29.1
PCB-17		1.8 ± 0.7	673	5.49	29.1
PCB-18		13 ± 5.0	1690	6.1	29.1
PCB-19		2.3 ± 0.7	235	5.03	29.1
PCB-20		5.5 ± 2.1	263	5.03	29.1
PCB-21		2.0 ± 0.5	813	5.66	29.1
PCB-22		5.7 ± 2.8	975	5.77	29.1
PCB-23		1.3 ± 0.5	391	5.4	29.1
PCB-24		15 ± 3.7	263	4.38	29.1
PCB-25		12 ± 2.8	20	3.51	38.33
PCB-26		2.8 ± 0.1	149	4.87	38.33
PCB-27		3.6 ± 1.3	1680	6.21	38.33
PCB-28		9.1 ± 0.8	13.9	3.62	38.33
PCB-29		0.45 ± 0.01	883	5.51	29.1
PCB-30		0.99 ± 0.30	121	5.14	29.1
PCB-31		0.94 ± 0.23	7.67	5.5	49.3

^1^MICs were determined against wild-type *M. tuberculosis* after 5 days growth (n ≥ 2). ^2^Human microsomal clearance.

SAR studies on the amine-side aryl ring designed to lower the cLogP revealed that the removal of the bromo group and a combination of a hydroxy and methoxy group to give **PCB-3** resulted in loss of activity (MIC > 20 µM), although clearance was improved. The cyclic ether **PCB-4** retained anti-tubercular activity with intermediate clearance. Limiting the substitution on this ring to a single *meta*-bromo substituent in **PCB-5** preserved the original biological activity (MIC = 4.9 µM). The *p*-bromo analog had similar activity (**PCB-6**, MIC = 5.4 µM), while the corresponding *o*-bromo compound was inactive (not shown, MIC > 20 µM). Replacement of the *m*-bromide by a –CF_3_ group (**PCB-7**, MIC = 3.7 µM) provided similar activity as well, but again with an increased clearance. Replacing the aryl ring with a bromopyridine to lower cLogP in **PCB-8** improved clearance but resulted in complete loss of activity (MIC > 20 µM). Similarly, various other polar ester, amide, and sulfonamide substituents on this ring each gave inactive analogs (MIC > 20 µM).

In studies on the right-side chain region, the two atom linkage to an arene gave the best activities. Attempted potency improvement through restricted rotation by cyclizing to the tetrahydronaphthyl (**PCB-9**) only maintained activity for the (*S*)-enantiomer (MIC = 6.9 µM) and increased microsomal clearance. To reduce oxidative liability and lower cLogP, the tetrahydronaphthyl was replaced by a quinazoline in **PCB-10**, which still had modest activity (MIC = 9.3 µM), and showed reasonable clearance. Other modifications to reduce lipophilicity with increased heteroatom count met with little success. Oxidation on the ethyl chain to give the alcohol **PCB-11** did provide lower clearance, but again with concomitant loss of activity (MIC > 20 µM). Further oxidation in **PCB-12** showed that the ketone was tolerated but this did not improve clearance. The oxadiazole amide surrogate **PCB-13** displayed both high clearance and loss of activity. Methylation of the amide nitrogen was examined to mitigate any clearance due to amide hydrolysis but provided the highly metabolized and inactive **PCB-14**.

The *m*-bromophenylethyl amide was chosen as a fixed right-hand moiety to explore the SAR of the left-hand aryl ring. Chlorophenyl rings in this region were tolerated, leading to a slight reduction, little change, or increase in activity moving from the *o*-, to *m*-, to *p*-regioisomer respectively (**PCB-15**, **PCB-16** and **PCB-17**) relative to **PCB-5**. In each case however, clearances increased, and the combined 2,4-dichloro **PCB-18** showed both very low stability and low antitubercular activity. Fluorophenyl rings showed an opposite regiochemical preference for activity relative to chloro substitution, with *para*-substitution being preferred (**PCB-19** and **PCB-20**). These analogs still showed very poor microsomal stability, even if improved over their chloro- counterparts. An *ortho*-bromo (**PCB-21)**, -trifluoromethyl (**PCB-22**), or -methyl group (**PCB-23**) on this ring provided varying changes in these measures, with the methyl being the most active of the series (MIC = 1.3 µM).

SAR studies of the central ring also demonstrated low tolerance for change. Attempting to circumvent CH oxidation with fluorines in difluoro **PCB-24** gave modest activity (MIC = 15 µM) but poor microsomal stability. An opposite strategy of increasing polarity with an oxetane ring did offer some improvement in clearance with **PCB-25**, which maintained modest activity as well (MIC = 12 µM).

Combining regional features from the above SAR provided mixed results. The fluorinated **PCB-26** delivered an expected boost in potency (MIC = 2.8 µM) over the parent **PCB-1** (MIC = 6.9 µM), but at the expense of clearance. The related analog **PCB-27** showed a phenyl could act as a bromide replacement in terms of activity, but with much higher clearance, consistent with its higher cLogP. Substitution of the cyclobutyl **PCB**-**7** with an oxetane in **PCB-28** again gave a loss in activity, but improved clearance. The combination of a left-hand *o*-toluyl group with the right-hand *m*-trifluorotoluyl group provided the most potent compound to date in the series with **PCB-29** (MIC = 0.45 µM), which unfortunately showed the predictable increase in clearance. Replacing the left-hand methyl with a *p*-fluoro group gave a slight reduction in activity, but significantly improved clearance with **PCB-30**. Use of this same fluorophenyl group with the dibromophenol group in the screening hit gave **PCB-31**, which displayed good anti-TB activity, and had one of the lowest human microsomal clearances in the series. Although, the clearance in mouse microsomes was several-fold higher, there was a several fold improvement over that of **PCB-2** (Cl_int,u_ 31.6 vs. 115 L/h/kg). The PK for **PCB-31** was therefore examined to determine if the better in vitro profile would translate to a better in vivo profile. Oral AUC did increase (259 vs. 58 ng h/mL at 30 mg/kg p.o.), but not sufficiently to make the compound a viable candidate. The analog also had a short IV half-life (0.20 h), and solubility-limited absorption, arising from high lipophilicity, highlighting the main liability of the series.

### MmpL3 is involved in the mechanism of action for both the 4PP and PCB series

Although our initial data did not suggest that MmpL3 was involved in the mechanism of action of the PCB, since there was no shift in MIC, we only used a single strain of *M. tuberculosis* and F255L is one of numerous MmpL3 mutations which can confer resistance to compounds^11–15^. Since we had seen a number of series with reduced activity against the *M. tuberculosis* strain carrying MmpL3_F255L_, we wanted to test MmpL3 as a target or mode of resistance for the PCB series further. We expanded our testing to include three additional strains with either G253E, Y252C, or Y252S mutations in MmpL3.

We tested 5 PCB analogs against these three strains. Interestingly, three of the mutant strains (G253E, Y252C, or Y252S alleles) were resistant to the five PCB analogs tested. In addition, three of the five analogs were significantly less active against the F255L mutant. We compared this profile to the 4PP series. All three of the 4PP hits from the primary screen had lower activity against the MmpL3_F255L_ mutant strain (Table 2). We tested one representative compound from the series (**4PP-1**) for activity. All of the mutant strains were resistant to the compounds, although the level of resistance varied from ~ 5 to 15 fold. (Table 5). These data suggest that both the 4PP and PCB series target MmpL3 directly, or that MmpL3 is involved in their mode of action and that MmpL3 mutations lead to resistance.

**Table 5:** Activity of PCB and 4PP analogs against *M. tuberculosis* MmpL3 mutant strains.

Chemotype	Molecule	Wild-type	MmpL3 F255L	MmpL3 G253E	MmpL3 Y252C	MmpL3 Y252S
		MIC (µM)	MIC (µM)	MIC (µM)	MIC (µM)	MIC (µM)
Phenylcyclobutane carboxamide	PCB-16	5.0	8.0	**75**	**43**	**50**
Phenylcyclobutanecarboxamide	PCB-17	1.8	**9.0**	**28**	**94**	**87**
Phenylcyclobutanecarboxamide	PCB-19	2.3	3	**20**	**45**	**35**
Phenylcyclobutanecarboxamide	PCB-21	2.0	**22**	**41**	**96**	** > 100**
Phenylcyclobutanecarboxamide	PCB-23	1.3	**8.0**	**27**	**83**	**82**
4-phenylpiperidine	4PP-1	6.3	**68**	**91**	**30**	**93**

Molecules from PCB and 4PP series were tested for activity against *M. tuberculosis* strains with mutations in MmpL3. MIC was determined against wild-type (WT) and four strains carrying MmpL3 alleles with F255L, Y252C, G253E, or Y252S. A significant loss of activity (≥ three-fold change) against mutant strain is noted in bold.

In order to determine if there were other targets or mechanism(s) of resistance, we isolated resistant mutants against **4PP-32**, **PCB-19** and **PCB-21**. We first determined the MIC on solid medium for the three compounds as 1.6 µM, 0.63 µM, and 1.25 µM respectively. We isolated resistant mutants on plates containing 5X MIC by plating ~ 10^8^ CFUs. The frequency of resistance was 7.35 × 10^−8^, 7.0 × 10^−8^ and 5.26 × 10^−8^, respectively. We sequenced MmpL3 in 28 confirmed mutants for **4PP-32**; all had mutations in MmpL3. We found twelve different mutations: Y252H, G253E, L576P, T588A, V643E, V643M, F644L, F644C, F644I, V646M, A700T, and A706T. We isolated 11 mutants against **PCB-21** and 4 against **PCB-19**; again, all had mutations in MmpL3. We found seven SNPs in the strains resistant to **PCB-21** (F255L, I292T, V646A, T670N, A677E, A678P, V684A) and one strain with an insertion at 708L. For **PCB-19**, we found three strains with the same SNP (T670I) and one strain with I292S. We did not isolate any strains without MmpL3 mutations. These data strongly suggest that MmpL3 is the primary target for both series, although we cannot exclude other mechanisms of resistance.

## Discussion

We screened a diverse, small molecule library from the AbbVie chemical collection for activity against *M. tuberculosis *in vitro. The screen had a hit rate of 1.3% and we identified 24 novel chemotypes that may be used as starting points for further development. The advantage of this screening approach is that all of these chemotypes have the ability to penetrate into *M. tuberculosis*. Of the 24 novel chemotypes described, 12 were singletons demonstrating the potential of diversity screening to identify novel starting points. We explored two of these series further.

We found that several of the *M. tuberculosis* actives identified in our screen had reduced activity in an MmpL3 F255L mutant strain. MmpL3 is a transmembrane protein involved in the transport of mycolic acids, essential components of the *M. tuberculosis* cell wall^16–18^. Many *M. tuberculosis* inhibitors identified in whole-cell screens, including several drugs currently in development for TB, are reported to have MmpL3-related mechanisms of resistance^13,19–25^. We hypothesize that MmpL3 is the target of both these series. The fact that some MmpL3 mutations conferred higher levels of resistance than others may reflect the mode and/or location of binding. Therefore, whilst testing molecules for activity against mutant strains offers valuable information, more comprehensive testing against a wider panel of mutants would provide more confidence in the preliminary mechanism of action suggested by these assays.

The initial hits from both series had undesirable physicochemical properties from a drug-development perspective, being highly lipophilic. Attempts to address this property with the addition of polar groups generally led to inactive analogs in both series. The 4PP series analogs had a good activity profile against *M. tuberculosis*, but there were liabilities with this series that we were unable to overcome in our initial exploration. Studies on the PCB series showed a similar trend. Microsomal clearances were tracked for these analogs to provide further information about the correlation of lipophilicity to their metabolic liability. Most attempts to reduce cLogP also reduced anti-tubercular activity. Slightly more progress was made in this series, leading to the sub-micromolar analog **PCB-31**, which showed acceptable microsomal clearance.

A number of MmpL3-targeting series have been described which are structurally distinct from the series presented here (reviewed in^21^. The indole carboxamides are the most advanced series in lead optimization which have demonstrated in vivo efficacy^26^. The spirocycle series had safety issues^27^, which could be resolved, but this led to compounds with reduced in vivo exposure (and no in vivo efficacy)^13^. Other series are earlier in the discovery process (hit-lead or lead generation) including: the tetrahydropyrazolo pyrimidine carboxamides which may target both MmpL3 and EchA6; the benzo-imidazoles which have poor solubility and high lipophilicity^28^; the benzothiazole amides which are highly lipophilic^29^; the adamantly urea AU1235, which contain a central urea liability^30^; and the pyrazole BM635 which has in vivo activity but also hERG activity as a liability^31^. Given the high value of the target and the high attrition rate in drug discovery/development (especially in developing new anti-tubercular agents) we propose that multiple series should be progressed simultaneously in order to meet the high need for new TB drugs.

In summary, we have identified several new chemotypes with activity against *M. tuberculosis* by phenotypic whole cell screening. Future work to explore each of these series systematically provide opportunities for the development of drugs to treat *M. tuberculosis* infections.

## Materials and methods

### Bacterial strains

*M. tuberculosis* H37Rv (London Pride, ATCC 25,618) expressing *Ds*Red was used for the primary screen^17^. *M. tuberculosis* isolates containing SNPs within genes of interest were originally isolated from wild-type ATCC 25,618^9–11^. *M. tuberculosis* was grown under aerobic conditions in Middlebrook 7H9 medium (Becton Dickinson) supplemented with 10% v/v OADC (oleic acid, albumin, dextrose, catalase), 0.05% w/v Tween 80 (7H9-Tw-OADC) or Middlebrook 7H10 agar supplemented with 10% v/v OADC.

### Whole cell screening of *M. tuberculosis*

High-throughput screening was performed as previously described^32,33^. Briefly, *M. tuberculosis* was exposed to compounds in 384-well plates at a starting OD of 0.06. Growth was measured by RFU (Ex560/Em590) after 5 days. Plates contained controls for maximum (2% DMSO) and minimum growth (2 µM rifampicin) and used to calculate % inhibition of growth.

### Minimum inhibitory concentration (MIC)

MICs were determined as described previously^32^. Briefly, *M. tuberculosis* was exposed to compounds as 10-point, two-fold serial dilutions in 96-well plates at a starting OD of 0.02. Growth was measured by RFU (Ex560/Em590) after 5 days. Plates contained controls for maximum (2% DMSO) and minimum growth (2 µM rifampicin) and used to calculate % growth. Dose response curves were generated with the Levenberg–Marquardt algorithm and the concentration required to inhibit growth by 90% was calculated (MIC).

### Cytotoxicity against HepG2 cells

HepG2 cells were seeded at 2000 cells per well in 384-well, black, clear bottom PDL coated plates in MEM medium supplemented with 10% FBS, 2 mM L-glutamine, 1 mM sodium pyruvate and incubated overnight at 37C, 5% CO2. Compounds were added and plates incubated for 72 h. Cell viability was measured using the CyQUANT® reagent and imaging on a ViewLux™ ultraHTS Microplate Imager. The IC_20_ and IC_50_ were calculated from dose response curves.

### Isolation and characterization of resistant mutants

Spontaneous resistant mutants were isolated by plating late-log-phase cultures of *M. tuberculosis* onto 7H10-OADC containing 5X the MIC of the given compound^12^. Colonies were streaked onto 7H10-OADC containing 5X the MIC of compound to confirm resistance. Genomic DNA was purified and the MmpL3 gene was PCR-amplified with primers Mf1 and Mr1 and sequenced using primers Mf1, Mf2, Mf3 and Mr2. Primer sequences were: Mf1, GCTGTTGACCTCGCGAGTGTG; Mf2, CAACGGCGAATGGAAGTGCTG; Mf3, CGCCCTGGAGCTGGATTCAATC; Mr1, GCTTTCTTCAACAATGCGGTGCAG; Mr2, AGCCGAACGCCAAGAACATCA.

### Microsomal metabolism

Compounds (0.5 µM) were incubated with 0.25 mg/mL liver microsomal proteins at pH 7.4 at 37 °C. Reactions were initiated with 0.5 µM NADPH and samples taken at 0, 5, 10, 15, 20 and 30 min. The reaction was stopped by addition of 95% ACN/5% MeOH containing an internal standard. Samples were combined in compound groups of six pre-sorted by mono molecular weight and analyzed by LC/MS/MS. The peak area ratios (analyte peak area/IS peak area) were converted to % remaining using the area ratio at time 0 as 100%. The. half-life (T ½) and intrinsic clearance (mCLint) were calculated.

### Mouse PK

AbbVie is committed to ensuring the humane care and use of laboratory animals in the company’s research and development programs. Our programs aim to exceed regulatory agency standards, and we are committed to the internationally accepted principles of the 3Rs (refinement, reduction, replacement). All animal studies were reviewed and approved by AbbVie's Institutional Animal Care and Use Committee in accordance with national regulations. All animal studies were conducted in an AAALAC accredited program where veterinary care and oversight was provided to ensure appropriate animal care. All data are reported in accordance with ARRIVE guidelines. The pharmacokinetic profiles of selected compounds were determined following 2 mg/kg IV or 30 mg/kg single oral doses in groups of three female Balb/c mice; mice were permitted free access to food and water. The IV dose was administered as a solution in DMSO: Tween 80: PEG-400: D5W (2:5:20:73, by volume); the oral dose was administered in an EtOH: Labrafil M1944 CS: Captex 300 (10:30:60, by volume) formulation. The dose volume was 10 mL/kg for both IV and oral administration. Serial blood samples (~ 40 µL) were obtained from each animal 0.1 (IV only), 0.25, 0.5, 1, 3, 6, 9, 12 and 24 h after dosing. Plasma concentrations of parent drug were determined by HPLC–MS/MS vs spiked standards prepared in mouse plasma. Peak plasma concentrations (C_max_) and the time to peak plasma concentration (T_max_) were determined directly from the plasma concentration data for each animal. The plasma concentration data were submitted to multi-exponential curve fitting using WinNonlin. The area under the plasma concentration–time curve from 0 to t hours after dosing (AUC_0-t_, t = time of the last measurable plasma concentration) was calculated using the linear trapezoidal rule. The residual area extrapolated to infinity, determined as the final measured plasma concentration (C_t_) divided by the terminal plasma elimination rate constant (β), was added to the AUC_0–t_ to produce the total area under the curve (AUC_0–∞_). The apparent total plasma clearance (CL_p_) was calculated by dividing the administered dose by the AUC_0–∞_. Half-life (t_1/2_) was determined with the following calculation: t_1/2_ = ln (2)/elimination rate constant. Oral bioavailability was calculated as the dose-normalized AUC_0-∞_ (AUC_0–∞_/D) from the oral dose divided by the corresponding dose normalized AUC_0–∞_ from intravenous dosing.

### Chemistry

All chemicals used were purchased pure from commercially available sources such as Sigma Aldrich, VWR, Fisher or other chemical vendors. ^1^H NMR (300 MHz) spectra were recorded on a Bruker Biospin NMR spectrometer. Thin layer chromatography was performed using Whatman silica gel 60 Å plates with florescent indicator and visualized using a UV lamp (254 nm) or KMnO_4_ stain. Flash chromatography was performed on Grace with GraceResolve Normal Phase disposable silica columns. High performance liquid chromatography (HPLC) was performed on a Gilson 322 HPLC pump with a Gilson UV/VIS-155 detector and a Phenomenex Gemini C18 column (10 µm, 250 mm × 10 mm). Liquid chromatography electrospray ionization mass spectroscopy (LC–MS/ESI–MS) were acquired on an Agilent LC/MSD-SL with an 1100 HPLC and G1956B mass spectrometer with a Phenomenex Gemini 5 μm C18 110 Å 50 × 3 mm column.

### Synthesis of 4PP analogs

**4PP-15**, **4PP-33**, **4PP-34**, and **4PP-41** were obtained from commercial sources.
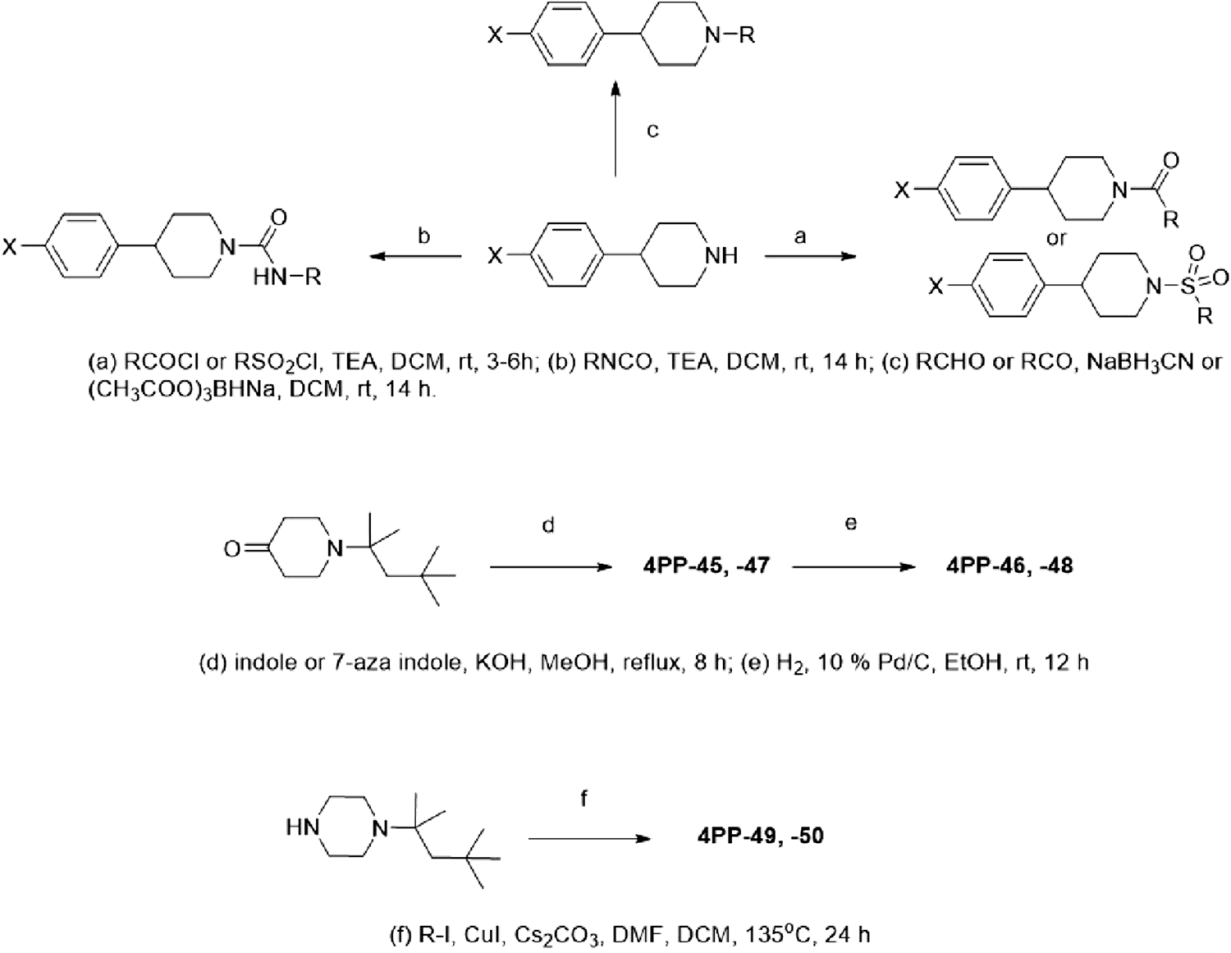


### Synthesis of PCB analogs


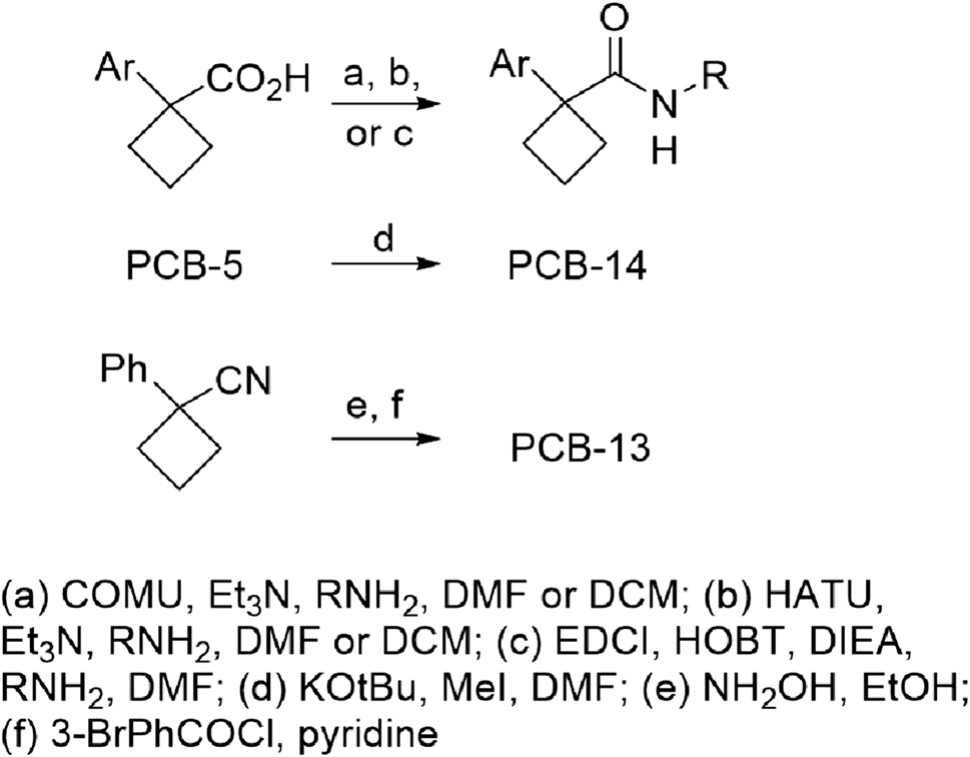


### General procedure 1 for synthesis of 4PPs

To a solution of the appropriate amine (2 mmol) was dissolved in DCM (10 ml) was added triethylamine (2.2 mmol), followed by the dropwise addition of the appropriate sulfonyl or acyl chloride (2 mmol). The reaction mixture was stirred at room temperature for 2–6 h. Saturated sodium chloride solution and ethyl acetate was added to the reaction mixture. The aqueous layer was extracted with ethyl acetate. The combined organic layer was dried over anhydrous sodium sulfate and concentrated in vacuum. The product was purified by flash column chromatography (SiO_2_; hexanes:EtOAc 1:0 to 0:1).

### General procedure 2

To a solution of the appropriate amine (1 mmol) and appropriate aldehyde or ketone (1 mmol) in DCM (5 mL) was added sodium cyanoborohydride (3 mmol), and stirred at room temperature for 14 h. Saturated sodium chloride solution and ethyl acetate was added to the reaction mixture. The aqueous layer was extracted with ethyl acetate. The combined organic layer was dried over anhydrous sodium sulfate and concentrated in vacuum. The product was purified by flash column chromatography (SiO_2_; hexanes:EtOAc 1:0 to 0:1).

### General procedure 3

To a solution of the appropriate amine (0.5 mmol) in DCE (5 mL) was added triethylamine (0.5 mmol) and stirred for 15 min at room temperature. The appropriate ketone (0.6 mmol) and sodium triacetoxyborohydride (1.5 mmol) was added to the reaction mixture and stirred at room temperature for 14 h Saturated sodium chloride solution and ethyl acetate was added to the reaction mixture. The aqueous layer was extracted with ethyl acetate. The combined organic layer was dried over anhydrous sodium sulfate and concentrated in vacuum. The product was purified by flash column chromatography (SiO_2_; DCM:MeOH 1:0 to 0:1).

### General procedure 4

To a solution of the appropriate amine (0.5 mmol) in DCM (4 mL) was added triethylamine (1 mmol), followed by the dropwise addition of the appropriate isocyanate (0.5 mmol). The reaction mixture was stirred at room temperature for 14 h. Saturated sodium chloride solution and ethyl acetate was added to the reaction mixture. The aqueous layer was extracted with ethyl acetate. The combined organic layer was dried over anhydrous sodium sulfate and concentrated in vacuum. The product was purified by flash column chromatography (SiO_2_; hexanes:EtOAc 1:0 to 0:1).

### General procedure 5

A mixture of amine (0.77 mmol), aldehyde (1.55 mmol), and 10% Pd/C (13 mg) in ethanol (5 ml) was stirred under H_2_ (1 atm) for 12 h, filtered, concentrated, and purified by prep HPLC.

#### 1-(1-Ethyl-cyclohexyl)-4-phenyl-piperidine (4PP-1)

Acquired externally: ^1^H NMR (400 MHz, DMSO_D_2_O) δ 7.33–7.26 (m, 2H), 7.25–7.21 (m, 2H), 7.20–7.15 (m, 1H), 3.01 (d, *J* = 11.3 Hz, 2H), 2.44 (ddt, *J* = 12.0, 7.6, 3.7 Hz, 1H), 2.29–2.16 (m, 2H), 1.76 (d, *J* = 12.4 Hz, 2H), 1.70–1.45 (m, 7H), 1.40 (q, *J* = 7.6 Hz, 2H), 1.36–1.19 (m, 5H), 0.80 (t, *J* = 7.5 Hz, 3H); MS (ESI +) *m/z* 272.2 [M + H]^+^.

#### 4-(4-tert-Butyl-phenyl)-1-(1-ethyl-cyclohexyl)-piperidine (4PP-2)

Acquired externally: ^1^H NMR (400 MHz, DMSO_D_2_O) δ 7.33–7.27 (m, 2H), 7.14 (d, *J* = 8.3 Hz, 2H), 3.00 (d, *J* = 11.3 Hz, 2H), 2.44–2.36 (m, 1H), 2.22 (t, *J* = 11.4 Hz, 2H), 1.75 (d, *J* = 12.8 Hz, 2H), 1.70–1.44 (m, 8H), 1.40 (q, *J* = 7.6 Hz, 2H), 1.26 (s, 13H), 0.80 (t, *J* = 7.6 Hz, 3H); MS (ESI +) *m/z* 328.3 [M + H]^+^.

#### 4-(4-*t*-Butylphenyl)-1-(cyclohexylmethyl)piperidine; 2,2,2-trifluoroacetic acid (4PP-3)

Method B (38%): ^1^H NMR (400 MHz, DMSO-*d*_6_) δ 7.35–7.29 (m, 2H), 7.15–7.09 (m, 2H), 3.53 (d, *J* = 12.2 Hz, 2H), 3.04–2.87 (m, 4H), 2.77–2.68 (m, 1H), 1.95–1.85 (m, 4H), 1.80–1.56 (m, 7H), 1.23 (s, 9H), 1.20–1.05 (m, 2H), 1.00–0.87 (m, 2H); MS (DCI) *m/z* 314.2 [M + H]^+^.

#### 1-Cyclohexylmethyl-4-phenyl-piperidine (4PP-4)

Acquired externally: ^1^H NMR (400 MHz, DMSO-*d*_6_) δ 7.32–7.10 (m, 5H), 2.93–2.82 (m, 2H), 2.43 (tt, *J* = 11.6, 4.0 Hz, 1H), 2.07 (d, *J* = 7.2 Hz, 2H), 1.91 (td, *J* = 11.6, 2.7 Hz, 2H), 1.77–1.54 (m, 9H), 1.55–1.45 (m, 1H), 1.25–1.04 (m, 3H), 0.89–0.74 (m, 2H); MS (DCI) *m/z* 258.1 [M + H]^+^.

#### 4-(4-(*t-*Butyl)phenyl)-1-(cyclohexylsulfonyl)piperidine (4PP-5)

Procedure 1. White powder (42% yield): ^1^H NMR (300 MHz, DMSO-d6) δ 7.32 (d, J = 8.3 Hz, 2H), 7.16 (d, J = 8.5 Hz, 2H), 3.72–3.76 (m, 2H), 3.02–3.23 (m, 3H), 2.86–3.06 (m, 1H), 1.88–2.13 (m, 2H), 1.68–1.91 (m, 4H), 1.29–1.70 (m, 8H), 1.26 (s, 9H); LCMS (ESI) m/z (M + H)^+^ 363.9.

#### (4-(4-(*t-*Butyl)phenyl)piperidin-1-yl)(cyclohexyl)methanone (4PP-6)

Procedure 1. White powder (54% yield): ^1^H NMR (300 MHz, Chloroform-d) δ 7.35 (d, J = 8.3 Hz, 2H), 7.15 (d, J = 8.3 Hz, 2H), 4.85–4.81 (m, 1H), 4.09–4.01 (m, 1H), 3.18–3.10 (m, 1H), 2.74–2.67 (m, 1H), 2.63–2.46 (m, 2H), 2.42–2.25 (m, 1H), 1.90–1.40 (m, 9H), 1.33 (s, 9H), 1.31–1.20 (m, 4H); LCMS–ESI (M + H)^+^: 328.0.

#### N-(*t-*Butyl)-4-(4-(tert-butyl)phenyl)piperidine-1-carboxamide (4PP-7)

Procedure 4. White powder (62% yield): ^1^H NMR (300 MHz, DMSO-d6) δ 7.31 (d, J = 8.2 Hz, 2H), 7.15 (d, J = 8.1 Hz, 2H), 5.76 (s, 1H), 4.09–4.04 (m, 2H), 2.75–2.56 (m, 3H), 1.72–1.68 (m, 2H), 1.57–1.36 (m, 2H), 1.26 (s, 18 H); LCMS–ESI (M + H)^+^: 317.0.

#### (4-(4-(*t*-Butyl)phenyl)piperidin-1-yl)(piperidin-1-yl)methanone (4PP-8)

Procedure 1. White powder (67% yield): ^1^H NMR (300 MHz, DMSO-d6) δ 7.31 (d, J = 7.9 Hz, 2H), 7.16 (d, J = 8.0 Hz, 2H), 3.63–3.67 (m, 2H), 3.06–3.19 (m, 4H), 2.72–2.88 (m, 2H), 2.54–2.70 (m, 1H), 1.73 (m, 2H), 1.39–1.65 (m, 8H), 1.26 (s, 9H); LCMS (ESI) m/z (M + H)^+^ 329.0.

#### 1-(Cyclohexylsulfonyl)-4-phenylpiperidine (4PP-9)

Procedure 1. White powder (45% yield): ^1^H NMR (300 MHz, DMSO-d6) δ 7.38–7.12 (m, 5H), 3.77–3.73 (m, 2H), 3.16–3.08 (m, 1H), 3.03–2.96 (m, 2H), 2.73–2.68 (m, 1H), 2.10–1.93 (m, 2H), 1.89–1.71 (m, 4H), 1.71–1.49 (m, 3H), 1.48–1.04 (m, 5H); LCMS–ESI (M + H)^+^: 307.9.

#### Cyclohexyl(4-phenylpiperidin-1-yl) methanone (4PP-10)

Procedure 1. White powder (59% yield): ^1^H NMR (300 MHz, Chloroform-d) δ 7.43–7.10 (m, 5H), 4.86–4.82 (m 1H), 4.09–4.05 (m, 1H), 3.25–2.97 (m, 1H), 2.87–2.69 (m, 1H), 2.69–2.37 (m, 2H), 2.04–1.40 (m, 11H), 1.43–1.11 (m, 3H); LCMS–ESI (M + H)^+^: 271.9.

#### N-(tert-butyl)-4-phenylpiperidine-1-carboxamide (4PP-11)

Procedure 4. White powder (69% yield): ^1^H NMR (300 MHz, DMSO-d6) δ 7.4–86.85 (m, 5H), 5.77 (broad s, 1H), 4.10–4.05 (m, 2H), 2.70–2.51 (m, 3H), 1.74–1.69 (m, 2H), 1.60–1.38 (m, 2H), 1.27 (s, 9H); LCMS–ESI (M + H)^+^: 261.0.

#### (4-Phenylpiperidin-1-yl)(piperidin-1-yl)methanone (4PP-12)

Procedure 1. White powder (62% yield): ^1^H NMR (300 MHz, Chloroform-d) δ 7.39–7.29 (m, 2H), 7.27–7.19 (m, 3H), 3.85–3.79 (m, 2H), 3.26–3.22 (m, 4H), 2.93–2.84 (m, 2H), 2.71–2.63 (m, 1H),), 1.88–1.84 (m, 2H), 1.80–1.66 (m, 2H), 1.66–1.49 (m, 6H); LCMS–ESI (M + H)^+^: 272.9.

#### N-cyclohexyl-4-phenylpiperidine-1-carboxamide (4PP-13)

Procedure 4. White powder (52% yield): ^1^H NMR (300 MHz, Chloroform-d) δ 7.34–7.13 (m, 5H), 6.15 (d, J = 7.7 Hz, 1H), 4.13–4.08 (m, 2H), 3.52–3.36 (m, 1H), 2.78–2.55 (m, 3H), 1.83–1.63 (m, 6H), 1.64–1.38 (m, 3H), 1.33–0.93 (m, 5H); LCMS–ESI (M + H)^+^: 287.2.

#### 3,3-Dimethyl-1-(4-phenylpiperidin-1-yl)butan-1-one (4PP-14)

Procedure 1. White powder (65% yield). 1H NMR (300 MHz, Chloroform-d) δ 7.39–7.29 (m, 2H), 7.28–7.13 (m, 3H), 4.91–4.87 (m, 1H), 4.12–4.07 (m, 1H), 3.20–3.11 (m, 1H), 2.79–2.71 (m, 1H), 2.67–2.63 (m, 1H), 2.34 (s, 2H), 1.94–1.90 (m, 2H), 1.76–1.54 (m, 2H), 1.10 (s, 9H); LCMS–ESI (M + H)^+^: 260.0.

#### 4-(4-*t*-Butyl-phenyl)-1-cyclohexyl-piperidine, hydrochloride (4PP-16)

Acquired externally: ^1^H NMR (400 MHz, DMSO-*d*_6_, 90 C) δ 7.36–7.28 (m, 2H), 7.16 (d, *J* = 7.9 Hz, 2H), 3.46 (d, *J* = 12.0 Hz, 2H), 3.06 (d, *J* = 12.7 Hz, 3H), 2.14 (d, *J* = 12.6 Hz, 4H), 1.98 (d, *J* = 14.1 Hz, 2H), 1.85 (d, *J* = 13.2 Hz, 2H), 1.68–1.59 (m, 1H), 1.48 (q, *J* = 11.7, 11.1 Hz, 2H), 1.38–1.28 (m, 2H), 1.28 (s, 9H), 1.16 (dddd, *J* = 16.5, 12.7, 8.1, 4.7 Hz, 1H); MS (ESI) *m/z* 330.4 [M + H]^+^.

#### 4-(4-(4-(*t*-Butyl)phenyl)piperidin-1-yl)-N,N-dimethylcyclohexan-1-amine (4PP-17)

Procedure 3. White powder (34% yield): ^1^H NMR (300 MHz, Chloroform-d) δ 7.34 (d, J = 8.3 Hz, 2H), 7.19 (d, J = 8.2 Hz, 2H), 3.12–3.08 (m, 2H), 2.54–2.31 (m, 2H), 2.26 (s, 6H), 2.14–2.00 (m, 2H), 1.99–1.63 (m, 8H), 1.59–1.36 (m, 5H), 1.33 (s, 9H); LCMS–ESI (M + H)^+^: 343.3.

#### Methyl 4-(4-(4-(tert-butyl)phenyl)piperidin-1-yl)cyclohexane-1-carboxylate (4PP-18)

Procedure 3. White powder (31% yield): ^1^H NMR (300 MHz, Chloroform-d) δ 7.34 (d, J = 8.8 Hz, 2H), 7.17 (d, J = 7.9 Hz, 2H), 3.71 (s, 3H), 3.36–3.15 (m, 2H), 2.90–2.69 (m, 1H), 2.69–2.39 (m, 4H), 2.35–2.18 (m, 2H), 2.03–1.96 (m, 2H), 1.96–1.81 (m, 4H), 1.67–1.52 (m, 4H), 1.32 (s, 9H); LCMS–ESI (M + H)^+^: 358.1.

#### 4-(4-(4-(tert-Butyl)phenyl)piperidin-1-yl)cyclohexane-1-carboxylic acid (4PP-19)

To a solution of **4PP-18** (0.050 g, 0.14 mmol) in DCM (5 mL) was added a solution of potassium hydroxide (0.100 g, 1.8 mmol) in 5 ml water, and the reaction mixture was refluxed for 14 h, neutralized, and extracted with ethyl acetate. The combined organic layer was dried over anhydrous sodium sulfate and concentrated in vacuum. The product was purified by flash column chromatography (SiO_2_; DCM:MeOH 1:0 to 0:1) to yield **4PP-19** as a white powder (22% yield): 1H NMR (300 MHz, DMSO-*d*_6_) δ 7.29 (d, J = 8.2 Hz, 2H), 7.14 (d, J = 9.0 Hz, 2H), 2.91–2.86 (m, 2H), 2.37–2.21 (m, 4H), 2.16–2.03 (m, 1H), 2.01–1.86 (m, 2H), 1.87–1.76 (m, 2H), 1.76–1.65 (m, 2H), 1.65–1.44 (m, 3H), 1.36–1.27 (m, 3H), 1.26 (s, 9H). LCMS–ESI (M + H)^+^: 344.1.

#### 4-[4-(4-*t*-Butyl-phenyl)-piperidin-1-yl]-cyclohexanone (4PP-20)

Acquired externally: ^1^H NMR (500 MHz, DMSO-*d*_6_) δ 7.32–7.26 (m, 2H), 7.18–7.11 (m, 2H), 2.98 (dt, *J* = 11.5, 3.2 Hz, 2H), 2.75 (tt, *J* = 10.0, 3.2 Hz, 1H), 2.48–2.31 (m, 3H), 2.30–2.20 (m, 4H), 2.00–1.91 (m, 2H), 1.81–1.70 (m, 4H), 1.60 (qd, *J* = 12.4, 3.7 Hz, 2H), 1.26 (s, 9H); MS (ESI) *m/z* 314.4 [M + H]^+^.

#### N,N-dimethyl-4-(4-phenylpiperidin-1-yl)cyclohexan-1-amine (4PP-21)

Procedure 2. White powder (30% yield): ^1^H NMR (300 MHz, DMSO-d6) δ 7.38–7.08 (m, 5H), 3.01–2.97 (m, 2H), 2.46–2.33 (m, 1H), 2.34–2.21 (m, 1H), 2.20–2.05 (m, 8H), 2.05–1.90 (m, 1H), 1.89–1.48 (m, 8H), 1.49–1.21 (m, 4H); LCMS–ESI (M + H)^+^: 287.2.

#### Methyl 4-(4-phenylpiperidin-1-yl)cyclohexane-1-carboxylate (4PP-22)

Procedure 2. White powder (18% yield): ^1^H NMR (300 MHz, DMSO-d6) δ 7.40 -7.03 (m, 5H), 3.60 (s, 3H), 2.96–2.88 (m, 2H), 2.49–2.36 (m, 2H), 2.37–2.09 (m, 3H), 2.07–1.88 (m, 2H), 1.87–1.68 (m, 3H), 1.67–41 (m, 5H), 1.41–1.19 (m, 2H); LCMS–ESI (M + H)^+^: 302.2.

#### 4-(4-Phenylpiperidin-1-yl)cyclohexan-1-ol (4PP-23)

Procedure 3. White powder (15% yield): ^1^H NMR (300 MHz, Chloroform-d) δ 7.39–7.09 (m, 5H), 3.68–3.47 (m, 1H), 3.21–2.94 (m, 2H), 2.60–2.40 (m, 1H), 2.43–2.22 (m, 3H), 2.16–2.02 (m, 1H), 2.02–1.48 (m, 9H), 1.49–1.17 (m, 3H); LCMS–ESI (M + H)^+^: 261.2.

#### 4-(4-(tert-Butyl)phenyl)-1-(tetrahydro-2H-pyran-4-yl)piperidine (4PP-24)

Procedure 3. White powder (26% yield): ^1^H NMR (300 MHz, Chloroform-d) δ 7.36 (d, J = 8.2 Hz, 2H), 7.19 (d, J = 8.2 Hz, 2H), 4.12–4.07 (m, 2H), 3.47 -3.40 (m, 2H), 3.26–3.22 (m, 2H), 2.85–2.69 (m, 1H), 2.66–2.33 (m, 3H), 2.04–1.84 (m, 6H), 1.85–1.60 (m, 2H), 1.33 (s, 9H); LCMS–ESI (M + H)^+^: 302.2.

#### 4-(4-(tert-Butyl)phenyl)-1'-methyl-1,4'-bipiperidine (4PP-25)

Procedure 3. White powder (32% yield): ^1^H NMR (300 MHz, Chloroform-d) δ 7.34 (d, J = 8.4 Hz, 2H), 7.18 (d, J = 8.3 Hz, 2H), 3.06–3.02 (m, 2H), 2.97–2.93 (m, 2H), 2.54–2.43 (m, 1H), 2.41–2.32 (m, 2H), 2.29 (s, 3H), 2.07—1.90 (m, 2H), 1.90–1.78 (m, 5H), 1.80–1.60 (m, 4H), 1.33 (s, 9H); LCMS–ESI (M + H)^+^: 315.2.

#### 4-Phenyl-1-(tetrahydro-2H-pyran-4-yl)piperidine (4PP-26)

Procedure 2. White powder (12% yield): ^1^H NMR (300 MHz, Chloroform-d) δ 7.14–7.42 (m, 5H), 4.10–4.05 (m, 2H), 3.46–3.38 (m, 2H), 3.14–3.10 (m, 2H), 2.66–2.42 (m, 2H), 2.34–2.25 (m, 2H), 1.98–1.57 (m, 8H); LCMS–ESI (M + H)^+^: 246.2.

#### 1'-Methyl-4-phenyl-1,4'-bipiperidine (4PP-27)

Procedure 2. White powder (12% yield): ^1^H NMR (300 MHz, DMSO-d6) δ 7.39–7.05 (m, 5H), 2.97–2.93 (m, 2H), 2.81–2.77 (m, 2H), 2.47–2.33 (m, 1H), 2.31–2.17 (m, 2H), 2.13 (s, 3H), 1.95–1.55 (m, 9H), 1.55–1.32 (m, 2H); LCMS–ESI (M + H)^+^: 259.2.

#### 4-(3-Bromophenyl)-1-(cyclohexylmethyl)piperidine; 2,2,2-trifluoroacetic acid (4PP-28)

Method B (67%): ^1^H NMR (400 MHz, DMSO-*d*_6_) δ 7.48–7.41 (m, 2H), 7.32 (t, *J* = 7.7 Hz, 1H), 7.28–7.22 (m, 1H), 3.57 (d, *J* = 12.2 Hz, 1H), 3.06–2.91 (m, 3H), 2.89–2.78 (m, 1H), 2.02–1.88 (m, 3H), 1.84–1.56 (m, 8H), 1.33–0.89 (m, 6H); MS (DCI) *m/z* 336.1 [M + H]^+^.

#### 4-(3-Chlorophenyl)-1-(cyclohexylmethyl)piperidine; 2,2,2-trifluoroacetic acid (4PP-29)

Method B (78%): ^1^H NMR (400 MHz, DMSO-*d*_6_) δ 7.36 (q, *J* = 7.1, 6.5 Hz, 1H), 7.33–7.25 (m, 2H), 7.19 (d, *J* = 7.6 Hz, 1H), 3.25 (d, *J* = 14.1 Hz, 1H), 3.05–2.89 (m, 4H), 2.85–2.77 (m, 1H), 2.02–1.86 (m, 4H), 1.84–1.55 (m, 7H), 1.31–1.06 (m, 3H), 1.01–0.87 (m, 2H); MS (DCI) *m/z* 292.1 [M + H]^+^.

#### 4-[1-(Cyclohexylmethyl)-4-piperidyl]benzonitrile; 2,2,2-trifluoroacetic acid (4PP-30)

Method 5 (63%): ^1^H NMR (400 MHz, DMSO-*d*_6_) δ 7.84–7.76 (m, 2H), 7.47–7.37 (m, 2H), 3.56 (d, *J* = 12.2 Hz, 2H), 3.04–2.96 (m, 2H), 2.96–2.85 (m, 3H), 2.00–1.87 (m, 4H), 1.80–1.55 (m, 6H), 1.32–1.05 (m, 3H), 1.00–0.87 (m, 2H); MS (DCI) *m/z* 283.1 [M + H]^+^.

#### 1-(Cyclohexylmethyl)-4-(1-naphthyl)piperidine; 2,2,2-trifluoroacetic acid (4PP-31)

Method 5 (27%): ^1^H NMR (400 MHz, DMSO-*d*_6_) δ 8.22 (d, *J* = 8.4 Hz, 1H), 7.96 (dd, *J* = 8.0, 1.6 Hz, 1H), 7.84 (d, *J* = 8.2 Hz, 1H), 7.65–7.49 (m, 3H), 7.42 (d, *J* = 7.1 Hz, 1H), 3.64 (d, *J* = 12.2 Hz, 2H), 3.30–3.16 (m, 2H), 2.99 (d, *J* = 6.8 Hz, 2H), 2.19–2.04 (m, 4H), 1.90–1.59 (m, 7H), 1.36–1.10 (m, 3H), 1.08–0.93 (m, 2H); MS (ESI) *m/z* 308.2 [M + H]^+^.

#### 4-[1-(Cyclohexylmethyl)-4-piperidyl]pyridine, 2,2,2-trifluoroacetic acid salt (4PP-32)

Method 5 (40%): ^1^H NMR (400 MHz, DMSO-*d*_6_) δ 9.26 (s, 1H), 8.84–8.76 (m, 2H), 7.79 (d, *J* = 5.9 Hz, 2H), 3.63 (d, *J* = 12.1 Hz, 2H), 3.22–3.01 (m, 3H), 3.00–2.92 (m, 2H), 2.19–1.88 (m, 4H), 1.90–1.57 (m, 6H), 1.37–1.07 (m, 3H), 1.05–0.87 (m, 2H); MS (DCI) *m/z* 259.1 [M + H]^+^.

#### 1'-(Cyclohexylmethyl)spiro[indene-1,4'-piperidine]; 2,2,2-trifluoroacetic acid (4PP-35)

Method C (43%): ^1^H NMR (400 MHz, DMSO-*d*_6_) δ 7.39–7.31 (m, 1H), 7.31–7.15 (m, 3H), 7.11 (d, *J* = 5.7 Hz, 1H), 6.87 (d, *J* = 5.6 Hz, 1H), 3.59 (d, *J* = 12.5 Hz, 2H), 3.23 (d, *J* = 12.5 Hz, 2H), 3.02 (t, *J* = 6.2 Hz, 2H), 2.45–2.34 (m, 2H), 1.89–1.56 (m, 6H), 1.36–1.08 (m, 5H), 1.05–0.90 (m, 2H); MS (DCI) *m/z* 282.1 [M + H]^+^.

#### 1-[1-(Cyclohexylmethyl)-4-phenyl-4-piperidyl]ethanone (4PP-36)

Method 5 (15%): ^1^H NMR (400 MHz, Chloroform-*d*) δ 7.38–7.28 (m, 4H), 7.27–7.22 (m, 1H), 2.71—2.62 (m, 2H), 2.50–2.39 (m, 2H), 2.22–2.11 (m, 2H), 2.11–2.00 (m, 4H), 1.91 (s, 3H), 1.77–1.62 (m, 5H), 1.46 (ddd, *J* = 10.9, 7.2, 3.5 Hz, 1H), 1.28–1.09 (m, 3H), 0.84 (q, *J* = 10.8 Hz, 2H); MS (DCI) *m/z* 300.2 [M + H]^+^.

#### 1-(Cyclohexylmethyl)-4-phenyl-piperidine-4-carbonitrile; 2,2,2-trifluoroacetic acid (4PP-37)

Method 5 (17%): ^1^H NMR (400 MHz, DMSO-d6) δ 7.54–7.44 (m, 4H), 7.41 (s, 1H), 3.71 (d, J = 13.0 Hz, 2H), 3.10 (s, 3H), 2.43 (s, 2H), 2.34 (d, J = 13.5 Hz, 2H), 1.66 (dt, J = 25.7, 13.7 Hz, 5H), 1.18 (dt, J = 33.4, 11.9 Hz, 3H), 0.95 (t, J = 11.8 Hz, 1H)); MS (DCI) *m/z* 310.1 [M + H]^+^.

#### *N*-[[1-(Cyclohexylmethyl)-4-phenyl-4-piperidyl]methyl]acetamide; 2,2,2-trifluoroacetic acid (4PP-38)

Method 5 (67%): ^1^H NMR (400 MHz, PYRIDINE-d_5_, 90 C) δ 7.40 (dd, J = 7.5, 1.8 Hz, 2H), 7.32 (t, J = 7.8 Hz, 2H), 7.19 (m, 1H), 3.59 (d, J = 6.3 Hz, 2H), 3.10–3.02 (m, 2H), 2.67–2.56 (m, 2H), 2.38 (d, J = 6.8 Hz, 2H), 2.32 (t, J = 5.6 Hz, 4H), 1.94 (s, 3H), 1.82–1.73 (m, 2H), 1.68–1.46 (m, 4H), 1.27–1.04 (m, 3H), 0.98–0.84 (m, 2H); MS (ESI) *m/z* 329.3 [M + H]^+^.

#### Methyl 1-(cyclohexylmethyl)-4-phenyl-piperidine-4-carboxylate; 2,2,2-trifluoroacetic acid (4PP-39)

Method 5 (37%): ^1^H NMR (400 MHz, DMSO-*d*_6_) δ 7.48–7.26 (m, 5H), 3.64 (s, 3H), 3.61–3.52 (m, 2H), 3.02–2.83 (m, 4H), 2.64 (d, *J* = 13.7 Hz, 2H), 2.05 (t, *J* = 12.5 Hz, 2H), 1.81–1.50 (m, 6H), 1.30- 1.05 (m, 3H), 0.98–0.82 (m, 2H); MS (DCI) *m/z* 316.1 [M + H]^+^.

#### [1-(Cyclohexylmethyl)-4-phenyl-4-piperidyl]methanol; 2,2,2-trifluoroacetic acid (4PP-40)

Method 5 (73%): ^1^H NMR (400 MHz, PYRIDINE, 90 C) δ 7.51–7.46 (m, 2H), 7.36 (dd, *J* = 8.5, 7.0 Hz, 2H), 7.26–7.19 (m, 1H), 3.77 (s, 2H), 3.17 (dt, *J* = 12.1, 4.5 Hz, 2H), 2.65 (ddd, *J* = 12.2, 9.6, 3.9 Hz, 2H), 2.52–2.38 (m, 6H), 1.80 (dd, *J* = 13.0, 3.6 Hz, 2H), 1.69 (ddq, *J* = 14.3, 7.3, 3.4 Hz, 1H), 1.61 (dq, *J* = 12.8, 4.1, 3.5 Hz, 2H), 1.53 (ddd, *J* = 11.1, 5.7, 3.0 Hz, 1H), 1.19 (qt, *J* = 12.1, 3.3 Hz, 2H), 1.09 (tt, *J* = 12.1, 3.1 Hz, 1H), 0.99–0.87 (m, 2H); MS (DCI) *m/z* 288.2 [M + H]^+^.

#### 1-(Benzo[d][1,3]dioxol-5-ylmethyl)-4-phenylpiperidine (4PP-42)

Procedure 2. White powder (22% yield): ^1^H NMR (300 MHz, Chloroform-d) δ 7.40–7.41 (m, 5H), 6.91 (s, 1H), 6.82–6.76 (m, 2H), 5.97 (s, 2H), 3.48 (s, 2H), 3.10—2.81 (m, 2H), 2.64–2.36 (m, 1H), 2.16—1.98 (m, 2H), 1.93–1.74 (m, 4H); LCMS–ESI (M + H)^+^: 295.9.

#### 4-(4-*t*-Butyl-phenyl)-1-(1,1,3,3-tetramethyl-butyl)-piperidine (4PP-43)

Acquired externally: ^1^H NMR (500 MHz, DMSO-*d*_6_) δ 7.32–7.25 (m, 2H), 7.16–7.09 (m, 2H), 3.08 (d, *J* = 11.0 Hz, 2H), 2.43–2.35 (m, 1H), 2.12 (t, *J* = 11.4 Hz, 2H), 1.73 (d, *J* = 12.4 Hz, 2H), 1.53 (dt, *J* = 12.7, 9.8 Hz, 2H), 1.43 (s, 2H), 1.25 (s, 9H), 1.06 (s, 6H), 1.02 (s, 9H); MS (ESI) *m/z* 330.4 [M + H]^+^.

#### 4-Phenyl-1-(1,1,3,3-tetramethyl-butyl)-piperidine (4PP-44)

Acquired externally: ^1^H NMR (400 MHz, DMSO_D_2_O) δ 7.29 (m, 2H), 7.25–7.14 (m, 3H), 3.09 (s, 2H), 2.44 (ddt, *J* = 11.8, 7.4, 3.6 Hz, 1H), 2.17 (t, *J* = 11.3 Hz, 2H), 1.76 (d, *J* = 12.2 Hz, 2H), 1.57 (qd, *J* = 12.3, 3.5 Hz, 2H), 1.43 (s, 2H), 1.09 (s, 6H), 1.01 (s, 9H); MS (DCI) *m/z* 274.1 [M + H]^+^.

#### 3-(1-(2,4,4-Trimethylpentan-2-yl)-1,2,3,6-tetrahydropyridin-4-yl)-1H-pyrrolo[2,3-b]pyridine (4PP-45)

1-(2,4,4-trimethylpentan-2-yl)piperidin-4-one (0.47 mmol) was dissolved in methanol followed by addition of 7-azaindole (1 mmol) and KOH (20 mmol). The reaction mixture was heated to reflux for 8 h. Saturated sodium chloride solution and ethyl acetate was added to the reaction mixture. The aqueous layer was extracted with ethyl acetate. The combined organic layer was dried over anhydrous sodium sulfate and concentrated in vacuum. The product was purified by flash column chromatography (SiO_2_; DCM:MeOH 1:0 to 0:1). Yellow powder (29% yield): ^1^H NMR (300 MHz, Chloroform-d) δ 8.34–8.30 (m, 1H), 8.25–8.21 (m, 1H), 7.32 (s, 1H), 7.12 (m, 1H), 6.24–6.20 (m, 1H), 3.47–3.32 (m, 2H), 2.86–2.82 (m, 2H), 2.66–2.51 (m, 2H), 1.55 (s, 2H), 1.25 (s, 6H), 1.06 (s, 9H); LCMS–ESI (M + H)^+^: 312.2.

#### 3-(1-(2,4,4-Trimethylpentan-2-yl)piperidin-4-yl)-1H-pyrrolo[2,3-b]pyridine (4PP-46)

To a solution of **4PP-45** (0.16 mmol) in ethanol (5 ml) was added10% palladium on charcoal (5 mg). The reaction mixture was degassed and stirred under H_2_ environment at room temperature for 12 h. The reaction mixture was filtered through celite and solvent was reduced concentrated in vacuum. The product was purified by flash column chromatography (SiO_2_; DCM:MeOH 1:0 to 0:1) to give **4PP-46** as a yellow powder (30% yield): 1H NMR (300 MHz, Chloroform-d) δ 8.77 (s, 1H), 8.30–8.27 (m, 1H), 7.99–7.96 (m, 1H), 7.10–7.05 (m, 2H), 3.29–3.03 (m, 2H), 2.89–2.67 (m, 1H), 2.38–2.21 (m, 2H), 2.08–1.93 (m, 2H), 1.85–1.75 (m, 2H), 1.49 (s, 2H), 1.17 (s, 6H), 1.06 (s, 9H); LCMS–ESI (M + H)^+^: 314.2.

#### 3-(1-(2,4,4-Trimethylpentan-2-yl)-1,2,3,6-tetrahydropyridin-4-yl)-1H-indole (4PP-47)

1-(2,4,4-trimethylpentan-2-yl)piperidin-4-one (0.47 mmol) was dissolved in methanol followed by addition of indole (1 mmol) and KOH (20 mmol). The reaction mixture was heated to reflux for 8 h. Saturated sodium chloride solution and ethyl acetate was added to the reaction mixture. The aqueous layer was extracted with ethyl acetate. The combined organic layer was dried over anhydrous sodium sulfate and concentrated in vacuum. The product was purified by flash column chromatography (SiO_2_; DCM:MeOH 1:0 to 0:1) to give **4PP-47** as a yellow powder (31% yield): ^1^H NMR (300 MHz, Chloroform-d) δ 8.16 (s, 1H), 7.98–7.78 (m, 1H), 7.40–7.37 (m, 1H), 7.25–7.07 (m, 3H), 6.25–6.21 (m, 1H), 3.44–3.40 (m, 2H), 2.86–2.82 (m, 2H), 2.65–2.47 (m, 2H), 1.55 (s, 2H), 1.25 (s, 6H), 1.06 (s, 9H); LCMS–ESI (M + H)^+^: 311.2.

#### 3-(1-(2,4,4-Trimethylpentan-2-yl)piperidin-4-yl)-1H-indole (4PP-48)

To a solution of **4PP-47** (0.16 mmol) in ethanol (5 ml) was added 10% palladium on charcoal (5 mg). The reaction mixture was degassed and stirred under H_2_ environment at room temperature for 12 h. The reaction mixture was filtered through celite and solvent was reduced concentrated in vacuum. The product was purified by flash column chromatography (SiO_2_; DCM:MeOH 1:0 to 0:1) to give **4PP-48** as a yellow powder (30% yield): 1H NMR (300 MHz, Chloroform-d) δ 8.00 (s, 1H), 7.67 (d, J = 7.8 Hz, 1H), 7.37 (d, J = 7.9 Hz, 1H), 7.25–7.06 (m, 2H), 6.98 (s, 1H), 3.21–3.17 (m, 2H), 2.87–2.78 (m, 1H), 2.35–2.28 (m, 2H), 2.09–2.05 (m, 2H), 1.93–1.67 (m, 2H), 1.51 (s, 2H), 1.19 (s, 6H), 1.06 (s, 9H); LCMS–ESI (M + H)^+^: 313.2.

#### 1-(4-(*t*-Butyl)phenyl)-4-(2,4,4-trimethylpentan-2-yl)piperazine (4PP-49)

A mixture of CuI (0.05 mmol), Cs_2_CO_3_ (0.98 mmol) was dissolved in DMF (1 mL), 1-(tert-butyl)-4-iodobenzene (1 mmol), and 1-(2,4,4-trimethylpentan-2-yl)piperazine (0.5 mmol) were added, and the mixture was stirred under air in a closed system at 135 °C for 24 h. The heterogeneous mixture was then cooled to room temperature and diluted with CH_2_Cl_2_. The resulting solution was directly filtered through Celite and the solvent removed under reduced pressure. The product was purified by flash column chromatography (SiO_2_; DCM:MeOH 1:0 to 0:1) to give **4PP-49** as a white powder (47% yield): 1H NMR (300 MHz, Chloroform-d) δ 7.41–7.20 (d, J = 8.6 Hz, 2H), 6.90 (d, J = 8.8 Hz, 2H), 3.25–3.03 (m, 4H), 2.87–2.58 (m, 4H), 1.47 (s, 2H), 1.32 (s, 9H), 1.14 (s, 6H), 1.04 (s, 9H); LCMS–ESI (M + H)^+^: 331.1.

#### 1-Phenyl-4-(2,4,4-trimethylpentan-2-yl)piperazine (4PP-50)

A mixture of CuI (0.05 mmol), Cs_2_CO_3_ (0.98 mmol) was dissolved in DMF (1 mL), 4-iodobenzene (1 mmol), and 1-(2,4,4-trimethylpentan-2-yl)piperazine (0.5 mmol) were added, and the mixture was stirred under air in a closed system at 135 °C for 24 h. The heterogeneous mixture was then cooled to room temperature and diluted with CH_2_Cl_2_. The resulting solution was directly filtered through Celite and the solvent removed under reduced pressure. The product was purified by flash column chromatography (SiO_2_; DCM:MeOH 1:0 to 0:1) to give **4PP-50** as a white powder (43% yield): 1H NMR (300 MHz, Chloroform-d) δ 7.22–7.34 (m, 2H), 7.00–6.89 (m, 2H), 6.90–6.78 (m, 1H), 3.18 (t, J = 5.0 Hz, 4H), 2.76 (t, J = 5.0 Hz, 4H), 1.47 (s, 2H), 1.15 (s, 6H), 1.04 (s, 9H); LCMS–ESI (M + H)^+^: 275.1.

#### 1-(Cyclohexylmethyl)-3-fluoro-4-phenyl-piperidine; 2,2,2-trifluoroacetic acid (4PP-51)

Method 5 (32%): ^1^H NMR (400 MHz, Chloroform-*d*) δ 7.37 (dd, *J* = 8.1, 6.5 Hz, 2H), 7.34–7.22 (m, 3H), 5.31–5.06 (m, 1H), 3.96 (d, *J* = 11.4 Hz, 1H), 3.75 (d, *J* = 11.9 Hz, 1H), 3.01 (dd, *J* = 0.12.7, 6.8 Hz, 1H), 2.92 (dd, *J* = 12.8, 6.3 Hz, 1H), 2.88–2.78 (m, 1H), 2.78–2.60 (m, 2H), 2.43 (q, *J* = 14.3 Hz, 1H), 2.09 (d, *J* = 8.5 Hz, 1H), 1.91–1.63 (m, 5H), 1.36–1.00 (m, 6H); MS (DCI) *m/z* 276.1 [M + H]^+^.

#### 4-(4-Chlorophenyl)-1-(cyclohexylmethyl)-3-fluoro-piperidine; 2,2,2-trifluoroacetic acid (4PP-52)

Method 5 (2%): ^1^H NMR (400 MHz, DMSO-*d*_6_, 120 C) δ 7.39–7.33 (m, 2H), 7.33–7.27 (m, 2H), 4.88–4.68 (m, 1H), 3.53–3.43 (m, 1H), 2.91–2.80 (m, 2H), 2.61 (br s, 3H), 1.99–1.57 (m, 8H), 1.35–1.13 (m, 3H), 1.05–0.92 (m, 2H); MS (DCI) *m/z* 310.1 [M + H]^+^.

#### 1-(Cyclohexylmethyl)-3-fluoro-4-(4-methoxyphenyl)piperidine; 2,2,2-trifluoroacetic acid (4PP-53)

Method 5 (37%): ^1^H NMR (400 MHz, DMSO-*d*_6_) δ 9.22 (s, 1H), 7.32–7.21 (m, 2H), 7.02–6.89 (m, 2H), 5.05–4.78 (m, 1H), 3.75 (s, 3H), 3.35–3.10 (m, 4H), 2.92 (t, *J* = 5.9 Hz, 2H), 2.40–2.30 (m, 1H), 2.13–1.97 (m, 1H), 1.88–1.53 (m, 6H), 1.37–1.05 (m, 3H), 1.03–0.82 (m, 2H); MS (DCI) *m/z* 306.1 [M + H]^+^.

### General procedure A for synthesis of PCBs

To a suspension of amine, 1-phenylcyclobutanecarboxylic acid (0.016 g, 0.093 mmol), and triethylamine (0.017 ml, 0.121 mmol) in CH_2_Cl_2_ (0.75 ml) at RT was added COMU (0.048 g, 0.112 mmol), and the mix was stirred overnight, diluted with EtOAc, washed with sat NaHCO_3_ and brine, dried (Na_2_SO_4_), and chromatographed (25% EtOAc/hept) to give the desired product.

### General procedure B

A solution of HATU (64 mg, 0.17 mmol, 1.2 eq) in DMA (1 mL) was added to a solution of the carboxylic acid (0.14 mmol, 1.0 eq) in DMA (166 uL), and stirred at room temperature for 5 min. The amine (0.4 M, 59 uL, 0.023 mmol, 1.5 eq) was then added, followed by neat iPr_2_NEt (122 uL, 0.7 mmol, 5.0 eq). The reaction was stirred at room temperature for 1 h, then purified directly via reverse phase HPLC to yield the title compound.

### General procedure C

To a solution of acid (0.12 mmol) and amine (1.2 eq) in 0.5 mL of DMF was added HOAT (1.2 eq) and EDCI (1.2 eq), and the mix was stirred at RT for 20 min., and iPr_2_NEt (1.2 eq) was added, stirred at RT overnight, diluted with MeOH and purified by prep HPLC to give the desired product.

#### N-(3-bromo-4-methoxyphenethyl)-1-phenylcyclobutane-1-carboxamide (PCB-1)

Acquired externally: ^1^H NMR (501 MHz, DMSO-*d*_6_) δ 7.53 (t, *J* = 5.7 Hz, 1H), 7.34–7.24 (m, 6H), 7.20 (ddt, *J* = 7.6, 6.3, 1.6 Hz, 1H), 6.97 (dd, *J* = 8.4, 2.1 Hz, 1H), 6.91 (d, *J* = 8.4 Hz, 1H), 3.79 (s, 3H), 3.19 (td, *J* = 6.8, 5.6 Hz, 2H), 2.69–2.52 (m, 4H), 2.35–2.26 (m, 2H), 1.82–1.65 (m, 2H); MS (ESI) *m/z* 388.4 [M + H]^+^.

#### N-(3,5-dibromo-4-hydroxyphenethyl)-1-phenylcyclobutane-1-carboxamide (PCB-2)

Acquired externally: ^1^H NMR (500 MHz, DMSO-*d*_6_) δ 7.33–7.22 (m, 5H), 7.22–7.15 (m, 1H), 6.96 (dd, *J* = 8.4, 2.1 Hz, 1H), 6.88 (d, *J* = 8.5 Hz, 1H), 3.76 (s, 3H), 3.17 (t, *J* = 6.9 Hz, 2H), 2.66–2.53 (m, 4H), 2.34–2.23 (m, 2H), 1.78–1.63 (m, 2H) ); MS (ESI) *m/z* 450 [M–H]^−^.

#### N-(4-hydroxy-3-methoxyphenethyl)-1-phenylcyclobutane-1-carboxamide (PCB-3)

Method A (23%): ^1^H NMR (400 MHz, Chloroform-*d*) δ 7.38–7.28 (m, 2H), 7.26–7.22 (m, 1H), 7.22–7.15 (m, 2H), 6.73 (d, *J* = 8.0 Hz, 1H), 6.55 (d, *J* = 2.0 Hz, 1H), 6.37 (dd, *J* = 8.1, 2.0 Hz, 1H), 5.47 (s, 1H), 5.07 (s, 1H), 3.82 (s, 3H), 3.37 (td, *J* = 6.8, 5.8 Hz, 2H), 2.83–2.71 (m, 2H), 2.59 (t, *J* = 6.8 Hz, 2H), 2.42 (ddd, *J* = 11.8, 9.3, 7.0 Hz, 2H), 2.15 (dddd, *J* = 16.2, 10.9, 9.0, 7.1 Hz, 1H), 1.85 (dddd, *J* = 14.9, 11.2, 9.3, 5.6 Hz, 1H); MS (DCI) *m/z* 326.3 [M + H]^+^.

#### N-(2-(benzo[d][1,3]dioxol-5-yl)ethyl)-1-phenylcyclobutane-1-carboxamide (PCB-4)

Method B (86%): ^1^H NMR (501 MHz, DMSO-*d*_6_) δ 7.51 (t, *J* = 5.6 Hz, 1H), 7.34–7.25 (m, 4H), 7.20 (ddt, *J* = 7.4, 6.1, 1.9 Hz, 1H), 6.72 (d, *J* = 7.8 Hz, 1H), 6.64 (d, *J* = 1.6 Hz, 1H), 6.47 (dd, *J* = 7.9, 1.7 Hz, 1H), 5.94 (s, 2H), 3.17 (td, *J* = 7.1, 5.7 Hz, 2H), 2.65 (dtd, *J* = 11.9, 5.6, 2.6 Hz, 2H), 2.55 (t, *J* = 7.0 Hz, 2H), 2.36–2.26 (m, 2H), 1.83–1.65 (m, 2H); MS (ESI) *m/z* 324.2 [M + H]^+^.

#### N-(3-bromophenethyl)-1-phenylcyclobutane-1-carboxamide (PCB-5)

Method C (67%): ^1^H NMR (400 MHz, DMSO-*d*_6_) δ 7.56 (t, *J* = 5.7 Hz, 1H), 7.41–7.24 (m, 6H), 7.24–7.17 (m, 1H), 7.15 (t, *J* = 7.7 Hz, 1H), 7.00 (dt, *J* = 7.6, 1.4 Hz, 1H), 3.23 (q, *J* = 6.6 Hz, 2H), 2.71–2.57 (m, 4H), 2.37–2.24 (m, 2H), 1.83–1.64 (m, 2H); MS (ESI) *m/z* 358.1 [M + H]^+^.

#### N-(4-bromophenethyl)-1-phenylcyclobutane-1-carboxamide (PCB-6)

Method A (19%): ^1^H NMR (501 MHz, Chloroform-*d*) δ 7.38–7.32 (m, 2H), 7.31–7.27 (m, 3H), 7.21–7.17 (m, 2H), 6.81–6.74 (m, 2H), 5.07 (s, 1H), 3.41–3.34 (m, 2H), 2.82–2.72 (m, 2H), 2.61 (t, *J* = 6.7 Hz, 2H), 2.48–2.38 (m, 3H), 2.14 (dtt, *J* = 11.0, 9.1, 7.2 Hz, 1H), 1.86 (dtt, *J* = 11.2, 9.3, 5.7 Hz, 1H); MS (DCI) *m/z* 375.0 [M + NH_4_]^+^.

#### 1-phenyl-N-(3-(trifluoromethyl)phenethyl)cyclobutane-1-carboxamide (PCB-7)

Method A (38%): ^1^H NMR (501 MHz, Chloroform-*d*) δ 7.44 (ddd, *J* = 7.7, 1.9, 1.0 Hz, 1H), 7.36–7.28 (m, 4H), 7.26–7.21 (m, 1H), 7.21–7.16 (m, 2H), 7.12 (dt, *J* = 7.0, 1.4 Hz, 1H), 5.04 (s, 1H), 3.42 (td, *J* = 6.7, 6.0 Hz, 2H), 2.82–2.70 (m, 4H), 2.48–2.37 (m, 2H), 2.15 (dtt, *J* = 11.1, 9.1, 7.2 Hz, 1H), 1.86 (dtt, *J* = 11.1, 9.3, 5.6 Hz, 1H); MS (ESI) *m/z* 348.1 [M + H]^+^.

#### N-(2-(4-bromopyridin-2-yl)ethyl)-1-phenylcyclobutane-1-carboxamide (PCB-8)

Method A (4%): ^1^H NMR (400 MHz, DMSO-*d*_6_) δ 8.32 (dd, *J* = 5.3, 0.6 Hz, 1H), 7.57 (t, *J* = 5.6 Hz, 1H), 7.47 (dd, *J* = 5.3, 1.9 Hz, 1H), 7.39 (d, *J* = 1.9 Hz, 1H), 7.35–7.23 (m, 4H), 7.23–7.15 (m, 1H), 2.81 (t, *J* = 6.8 Hz, 2H), 2.63 (dtd, *J* = 14.4, 5.7, 2.5 Hz, 2H), 2.36–2.24 (m, 2H), 1.83–1.64 (m, 2H); LCMS (ESI) *m/z* 358.7 [M + H]^+^.

#### (S)-N-(7-bromo-1,2,3,4-tetrahydronaphthalen-2-yl)-1-phenylcyclobutane-1-carboxamide (PCB-9)

Method A (65%): ^1^H NMR (400 MHz, Chloroform-*d*) δ 7.33 (t, *J* = 7.5 Hz, 2H), 7.29–7.16 (m, 4H), 7.10 (d, *J* = 2.1 Hz, 1H), 6.87 (d, *J* = 8.2 Hz, 1H), 5.07 (d, *J* = 7.9 Hz, 1H), 4.26–4.07 (m, 1H), 3.03–2.64 (m, 4H), 2.61–2.31 (m, 4H), 2.23–2.06 (m, 1H), 1.89 (dddd, *J* = 11.1, 9.2, 7.6, 4.7 Hz, 2H), 1.55 (dtd, *J* = 12.8, 8.9, 5.8 Hz, 1H); MS (DCI) *m/z* 384.0 [M + H]^+^.

#### N-(5-bromoquinazolin-2-yl)-1-phenylcyclobutane-1-carboxamide (PCB-10)

Method C (11%): ^1^H NMR (400 MHz, Chloroform-*d*) δ 9.47 (s, 1H), 7.88 (d, *J* = 8.1 Hz, 1H), 7.81 (s, 1H), 7.75–7.60 (m, 2H), 7.43 (dd, *J* = 3.8, 1.1 Hz, 4H), 7.37–7.30 (m, 1H), 3.02 (ddd, *J* = 11.5, 8.9, 5.3 Hz, 2H), 2.67–2.50 (m, 2H), 2.26 (dt, *J* = 18.1, 8.6 Hz, 1H), 1.97 (ddt, *J* = 14.6, 9.3, 4.9 Hz, 1H); MS (DCI) *m/z* 382.0 [M + H]^+^.

#### N-(2-(benzo[d][1,3]dioxol-5-yl)-2-hydroxyethyl)-1-phenylcyclobutane-1-carboxamide (PCB-11)

Method A (47%): ^1^H NMR (400 MHz, Chloroform-*d*) δ 7.41–7.31 (m, 2H), 7.31–7.21 (m, 3H), 6.73–6.66 (m, 2H), 6.63 (dd, *J* = 8.0, 1.6 Hz, 1H), 5.93 (d, *J* = 1.0 Hz, 2H), 5.41 (s, 1H), 4.65 (dd, *J* = 7.0, 3.7 Hz, 1H), 3.50 (ddtd, *J* = 13.9, 7.1, 3.6, 1.1 Hz, 1H), 3.25 (dddd, *J* = 13.7, 6.7, 5.5, 1.0 Hz, 1H), 2.86–2.75 (m, 2H), 2.46 (tdd, *J* = 9.4, 7.1, 2.8 Hz, 2H), 2.23–2.08 (m, 1H), 1.95–1.81 (m, 1H); MS (ESI) *m/z* 338.2 [M-H]^-^.

#### N-(2-(3-bromophenyl)-2-oxoethyl)-1-phenylcyclobutane-1-carboxamide (PCB-12)

Method A (40%): ^1^H NMR (501 MHz, Chloroform-*d*) δ 8.04 (t, *J* = 1.8 Hz, 1H), 7.82 (ddd, *J* = 7.8, 1.8, 1.0 Hz, 1H), 7.71 (ddd, *J* = 7.9, 1.9, 1.0 Hz, 1H), 7.45–7.39 (m, 2H), 7.39–7.33 (m, 3H), 7.30 (ddt, *J* = 8.6, 6.7, 1.5 Hz, 1H), 6.17 (s, 1H), 4.62 (d, *J* = 4.4 Hz, 2H), 2.92–2.83 (m, 2H), 2.54 (tdd, *J* = 9.5, 7.1, 2.5 Hz, 2H), 2.17 (dtt, *J* = 11.2, 9.1, 7.2 Hz, 1H), 1.92 (dtt, *J* = 11.1, 9.2, 5.6 Hz, 1H); MS (DCI) *m/z* 389.1 [M + NH_4_]^+^.

#### 5-(3-bromophenyl)-3-(1-phenylcyclobutyl)-1,2,4-oxadiazole (PCB-13)

A solution of 1-phenylcyclobutanecarbonitrile (0.407 g, 2.59 mmol) in Ethanol (3.2 ml) was treated with 50% hydroxylamine (0.79 ml, 13 mmol). heated overnight at 80 °C, concentrated, washed with water and heptanes, filtered, triturated with EtOAc, filtered, and concentrated again to yield crude N-hydroxy-1-phenylcyclobutanecarboximidamide (0.396 g, 2.08 mmol, 80% yield).

A mixture of crude N-hydroxy-1-phenylcyclobutanecarboximidamide (0.0495 g, 0.260 mmol) and 3-bromobenzoyl chloride (0.034 ml, 0.26 mmol) in Pyridine (2 ml) was stirred at RT for 15 min heated to 105 °C for 18 h, concentrated, washed with NaHCO_3_, extracted using DCM, dried (Na_2_SO_4_), filtered, and purified by prep HPLC to give 5-(3-bromophenyl)-3-(1-phenylcyclobutyl)-1,2,4-oxadiazole (0.0258 g, 0.073 mmol, 28% yield): ^1^H NMR (501 MHz, Chloroform-*d*) δ 8.25 (t, *J* = 1.7 Hz, 1H), 8.02 (ddd, *J* = 7.8, 1.6, 1.1 Hz, 1H), 7.67 (ddd, *J* = 8.0, 2.0, 1.0 Hz, 1H), 7.42–7.33 (m, 5H), 7.26–7.21 (m, 1H), 3.03–2.94 (m, 2H), 2.82–2.72 (m, 2H), 2.21 (dp, *J* = 11.3, 8.7 Hz, 1H), 2.01 (dtt, *J* = 11.3, 9.0, 4.3 Hz, 1H); MS (DCI) *m/z* 372.0 [M + NH_4_]^+^.

#### N-(3-bromophenethyl)-N-methyl-1-phenylcyclobutane-1-carboxamide (PCB-14)

To a mixture of **PCB-5** (0.0642 g, 0.179 mmol) and potassium tert butoxide (0.080 g, 0.72 mmol) in DMF (3.0 mL) was added methyl iodide (0.045 mL, 0.72 mmol), and the mix was stirred at RT for three days. diluted with water and ethyl acetate, washed with brine, re-extracted with EtOAc, dried (Na_2_SO_4_), and purified by prep HPLC to give **PCB-14** (0.0175 g, 0.047 mmol, 26% yield): ^1^H NMR (400 MHz, DMSO-*d*_6_) δ 7.42–7.27 (m, 6H), 7.27–7.16 (m, 2H), 7.10 (d, *J* = 7.8 Hz, 1H), 2.73 (tdd, *J* = 9.8, 7.7, 2.2 Hz, 2H), 2.66 (d, *J* = 8.6 Hz, 2H), 2.56 (s, 3H), 2.37–2.26 (m, 2H), 1.91 (dp, *J* = 10.5, 8.2 Hz, 1H), 1.85–1.72 (m, 1H); MS (DCI) *m/z* 374.0 [M + H]^+^.

#### N-(3-bromophenethyl)-1-(4-chlorophenyl)cyclobutane-1-carboxamide (PCB-15)

Method C (61%): ^1^H NMR (400 MHz, Chloroform-*d*) δ 7.39–7.24 (m, 3H), 7.20–7.11 (m, 3H), 7.07 (t, *J* = 7.8 Hz, 1H), 6.84 (dt, *J* = 7.7, 1.3 Hz, 1H), 5.02 (s, 1H), 3.39 (q, *J* = 6.4 Hz, 2H), 2.84–2.70 (m, 2H), 2.65 (t, *J* = 6.6 Hz, 2H), 2.37 (tdd, *J* = 9.2, 7.1, 2.4 Hz, 2H), 2.13 (dtt, *J* = 10.9, 8.9, 7.0 Hz, 1H), 1.92–1.80 (m, 1H); MS (ESI) *m/z* 394.0 [M + H]^+^.

#### N-(3-bromophenethyl)-1-(3-chlorophenyl)cyclobutane-1-carboxamide (PCB-16)

Method C (66%): ^1^H NMR (400 MHz, Chloroform-*d*) δ 7.36–7.21 (m, 3H), 7.19 (dd, *J* = 4.3, 2.1 Hz, 2H), 7.13–7.03 (m, 2H), 6.85 (dt, *J* = 7.8, 1.3 Hz, 1H), 5.04 (s, 1H), 3.41 (q, *J* = 6.4 Hz, 2H), 2.82–2.70 (m, 2H), 2.66 (t, *J* = 6.6 Hz, 2H), 2.47–2.33 (m, 2H), 2.13 (dtt, *J* = 11.0, 8.9, 7.2 Hz, 1H), 1.91–1.79 (m, 1H); MS (ESI) *m/z* 393.9 [M + H]^+^.

#### N-(3-bromophenethyl)-1-(2-chlorophenyl)cyclobutane-1-carboxamide (PCB-17)

Method C (58%): ^1^H NMR (400 MHz, Chloroform-*d*) δ 7.35 (dd, *J* = 7.6, 1.4 Hz, 1H), 7.32–7.19 (m, 4H), 7.17 (d, *J* = 1.9 Hz, 1H), 7.05 (t, *J* = 7.8 Hz, 1H), 6.92 (d, *J* = 7.6 Hz, 1H), 5.09 (d, *J* = 6.2 Hz, 1H), 3.43 (q, *J* = 6.5 Hz, 2H), 2.83 (ddt, *J* = 12.6, 9.0, 3.1 Hz, 2H), 2.67 (t, *J* = 6.7 Hz, 2H), 2.47 (dt, *J* = 12.3, 9.4 Hz, 2H), 2.26 (dp, *J* = 11.2, 8.8 Hz, 1H), 1.89–1.75 (m, 1H); MS (ESI) *m/z* 394.0 [M + H]^+^.

#### N-(3-bromophenethyl)-1-(2,4-dichlorophenyl)cyclobutane-1-carboxamide (PCB-18)

Method C (48%): ^1^H NMR (400 MHz, Chloroform-*d*) δ 7.35 (d, *J* = 2.0 Hz, 1H), 7.32 (ddd, *J* = 7.9, 2.0, 1.0 Hz, 1H), 7.28–7.20 (m, 2H), 7.19 (d, *J* = 3.7 Hz, 1H), 7.07 (t, *J* = 7.8 Hz, 1H), 6.92 (dt, *J* = 7.6, 1.3 Hz, 1H), 5.08 (d, *J* = 6.2 Hz, 1H), 3.49–3.39 (m, 2H), 2.81 (dddd, *J* = 13.1, 6.6, 3.8, 1.7 Hz, 2H), 2.69 (t, *J* = 6.7 Hz, 2H), 2.49–2.35 (m, 2H), 2.31–2.19 (m, 1H), 1.81 (dtt, *J* = 11.0, 9.4, 4.2 Hz, 1H); MS (ESI) *m/z* 427.9 [M + H]^+^.

#### N-(3-bromophenethyl)-1-(4-fluorophenyl)cyclobutane-1-carboxamide (PCB-19)

Method C (63%): ^1^H NMR (400 MHz, Chloroform-*d*) δ 7.37–7.29 (m, 1H), 7.22–7.12 (m, 3H), 7.12–6.97 (m, 3H), 6.92–6.83 (m, 1H), 5.01 (s, 1H), 3.40 (q, *J* = 6.4 Hz, 2H), 2.76 (dtd, *J* = 11.7, 5.7, 2.4 Hz, 2H), 2.65 (t, *J* = 6.6 Hz, 2H), 2.38 (tdd, *J* = 9.3, 7.0, 2.4 Hz, 2H), 2.13 (dtt, *J* = 11.2, 9.1, 7.0 Hz, 1H), 1.92–1.81 (m, 1H); MS (ESI) *m/z* 376.1 [M + H]^+^.

#### N-(3-bromophenethyl)-1-(2-fluorophenyl)cyclobutane-1-carboxamide (PCB-20)

Method C (80%): ^1^H NMR (400 MHz, Chloroform-*d*) δ 7.31 (dt, *J* = 8.1, 1.5 Hz, 1H), 7.28–7.21 (m, 2H), 7.20 (t, *J* = 1.6 Hz, 1H), 7.16–7.10 (m, 1H), 7.07 (t, *J* = 7.8 Hz, 1H), 7.01 (ddd, *J* = 11.0, 8.1, 1.3 Hz, 1H), 6.93 (dt, *J* = 7.7, 1.3 Hz, 1H), 5.35 (s, 1H), 3.43 (q, *J* = 6.5 Hz, 2H), 2.85–2.73 (m, 2H), 2.68 (t, *J* = 6.7 Hz, 2H), 2.47 (qd, *J* = 9.0, 2.5 Hz, 2H), 2.20 (dp, *J* = 11.1, 8.8 Hz, 1H), 1.86 (dtt, *J* = 11.0, 9.2, 4.5 Hz, 1H); MS (ESI) *m/z* 376.0 [M + H]^+^.

#### N-(3-bromophenethyl)-1-(2-bromophenyl)cyclobutane-1-carboxamide (PCB-21)

Method C (50%): ^1^H NMR (400 MHz, Chloroform-*d*) δ 7.59–7.51 (m, 1H), 7.37–7.26 (m, 3H), 7.21–7.10 (m, 2H), 7.05 (t, *J* = 7.8 Hz, 1H), 6.93 (dd, *J* = 7.6, 1.4 Hz, 1H), 5.03 (s, 1H), 3.43 (q, *J* = 6.4 Hz, 2H), 2.86 (ddd, *J* = 12.8, 9.1, 3.9 Hz, 2H), 2.68 (t, *J* = 6.7 Hz, 2H), 2.48 (dt, *J* = 12.2, 9.2 Hz, 2H), 2.27 (dp, *J* = 11.1, 8.7 Hz, 1H), 1.80 (dddd, *J* = 14.0, 9.6, 7.3, 4.4 Hz, 1H); MS (ESI) *m/z* 437.9 [M + H]^+^.

#### N-(3-bromophenethyl)-1-(2-(trifluoromethyl)phenyl)cyclobutane-1-carboxamide (PCB-22)

Method A (34%): ^1^H NMR (400 MHz, Chloroform-*d*) δ 7.69–7.63 (m, 1H), 7.53 (td, *J* = 7.7, 1.4 Hz, 1H), 7.39 (tt, *J* = 7.6, 1.1 Hz, 1H), 7.34–7.27 (m, 2H), 7.13 (t, *J* = 1.8 Hz, 1H), 7.05 (t, *J* = 7.8 Hz, 1H), 6.90 (dt, *J* = 7.6, 1.4 Hz, 1H), 4.90 (s, 1H), 3.41 (q, *J* = 6.5 Hz, 2H), 2.83–2.71 (m, 2H), 2.69–2.52 (m, 4H), 2.45–2.31 (m, 1H), 1.79 (dtt, *J* = 10.7, 9.6, 2.9 Hz, 1H); MS (DCI) *m/z* 443.0 [M + NH_4_]^+^.

#### N-(3-bromophenethyl)-1-(o-tolyl)cyclobutane-1-carboxamide (PCB-23)

Method C (71%): ^1^H NMR (400 MHz, Chloroform-*d*) δ 7.29 (ddd, *J* = 7.9, 2.0, 1.0 Hz, 1H), 7.22–7.16 (m, 3H), 7.16–7.10 (m, 2H), 7.04 (t, *J* = 7.8 Hz, 1H), 6.84 (dt, *J* = 7.7, 1.3 Hz, 1H), 4.97 (t, *J* = 5.6 Hz, 1H), 3.39 (td, *J* = 6.7, 5.9 Hz, 2H), 2.74 (dddt, *J* = 11.5, 6.3, 4.6, 2.6 Hz, 2H), 2.62 (t, *J* = 6.8 Hz, 2H), 2.47 (qd, *J* = 9.2, 2.0 Hz, 2H), 2.31 (dp, *J* = 10.8, 8.9 Hz, 1H), 2.04 (d, *J* = 0.6 Hz, 3H), 1.81 (dtt, *J* = 10.9, 9.6, 3.9 Hz, 1H); MS (ESI) *m/z* 374.0 [M + H]^+^.

#### N-(3-bromophenethyl)-3,3-difluoro-1-phenylcyclobutane-1-carboxamide (PCB-24)

Method C (62%): ^1^H NMR (400 MHz, Chloroform-*d*) δ 7.39 (td, *J* = 6.9, 1.2 Hz, 2H), 7.35–7.28 (m, 2H), 7.25–7.16 (m, 2H), 7.12 (d, *J* = 1.8 Hz, 1H), 7.10–7.02 (m, 1H), 6.84 (dt, *J* = 7.6, 1.4 Hz, 1H), 5.10 (s, 1H), 3.53–3.33 (m, 4H), 3.04–2.90 (m, 2H), 2.63 (t, *J* = 6.6 Hz, 2H); MS (ESI) *m/z* 396.0 [M + H]^+^.

#### N-(3-bromophenethyl)-3-phenyloxetane-3-carboxamide (PCB-25)

Method A (56%): ^1^H NMR (400 MHz, Chloroform-*d*) δ 7.43—7.37 (m, 2H), 7.36–7.28 (m, 2H), 7.20–7.02 (m, 4H), 6.88 (dd, *J* = 7.7, 1.5 Hz, 1H), 5.22 (d, *J* = 5.7 Hz, 2H), 5.18 (s, 1H), 4.93 (d, *J* = 5.7 Hz, 2H), 3.44 (q, *J* = 6.4 Hz, 2H), 2.67 (t, *J* = 6.7 Hz, 2H); MS (DCI) *m/z* 379.0 [M + NH_4_]^+^.

#### N-(3-bromo-4-methoxyphenethyl)-1-(4-fluorophenyl)cyclobutane-1-carboxamide (PCB-26)

Method A (60%): ^1^H NMR (400 MHz, Chloroform-*d*) δ 7.20–7.14 (m, 3H), 7.07–6.95 (m, 2H), 6.81 (dd, *J* = 8.4, 2.2 Hz, 1H), 6.72 (d, *J* = 8.4 Hz, 1H), 4.98 (s, 1H), 3.87 (s, 3H), 3.37 (q, *J* = 6.4 Hz, 2H), 2.76 (ddd, *J* = 11.7, 9.1, 5.6 Hz, 2H), 2.60 (t, *J* = 6.6 Hz, 2H), 2.44–2.32 (m, 2H), 2.21–2.07 (m, 1H), 1.92–1.77 (m, 1H); MS (DCI) *m/z* 425.0 [M + NH_4_]^+^.

#### 1-(2-chlorophenyl)-N-(2-(6-methoxy-[1,1'-biphenyl]-3-yl)ethyl)cyclobutane-1-carboxamide (PCB-27)

Acquired externally: ^1^H NMR (501 MHz, DMSO-*d*_6_) δ 7.46–7.36 (m, 5H), 7.31 (td, *J* = 7.3, 1.6 Hz, 2H), 7.28 (dd, *J* = 7.9, 1.5 Hz, 1H), 7.22 (ddd, *J* = 7.9, 7.2, 1.7 Hz, 1H), 7.06–7.00 (m, 2H), 6.97–6.92 (m, 1H), 6.85 (t, *J* = 5.7 Hz, 1H), 3.71 (s, 3H), 3.29–3.22 (m, 2H), 2.72–2.60 (m, 4H), 2.34 (dt, *J* = 12.4, 9.3 Hz, 2H), 1.95 (dp, *J* = 10.8, 8.7 Hz, 1H), 1.69 (dddd, *J* = 13.4, 10.7, 9.1, 4.2 Hz, 1H); MS (ESI) *m/z* 420.3 [M + H]^+^.

#### 3-phenyl-N-(3-(trifluoromethyl)phenethyl)oxetane-3-carboxamide (PCB-28)

Method B (45%): ^1^H NMR (400 MHz, DMSO-*d*_6_) δ 7.55–7.45 (m, 2H), 7.41 (tt, *J* = 7.8, 0.7 Hz, 1H), 7.37–7.21 (m, 6H), 4.97 (d, *J* = 6.3 Hz, 2H), 4.73 (d, *J* = 6.4 Hz, 2H), 3.33 (t, *J* = 6.8 Hz, 2H), 2.78 (t, *J* = 6.8 Hz, 3H); MS (APCI) *m/z* 349.8 [M + H]^+^.

#### 1-(o-tolyl)-N-(3-(trifluoromethyl)phenethyl)cyclobutane-1-carboxamide (PCB-29)

Method B (26%): ^1^H NMR (400 MHz, DMSO-*d*_6_) δ 7.53–7.45 (m, 1H), 7.45–7.35 (m, 2H), 7.29–7.19 (m, 2H), 7.14 (td, *J* = 7.5, 1.7 Hz, 1H), 7.09 (td, *J* = 7.3, 1.6 Hz, 1H), 7.05–7.00 (m, 1H), 3.27 (t, *J* = 6.8 Hz, 2H), 2.72 (t, *J* = 6.8 Hz, 2H), 2.65–2.53 (m, 2H), 2.29 (qd, *J* = 9.2, 2.6 Hz, 2H), 1.98 (s, 3H), 1.88–1.76 (m, 1H), 1.70—1.60 (m, 1H); MS (APCI) *m/z* 362.2 [M + H]^+^.

#### 1-(4-fluorophenyl)-N-(3-(trifluoromethyl)phenethyl)cyclobutane-1-carboxamide (PCB-30)

Method B (77%): ^1^H NMR (400 MHz, DMSO-*d*_6_) δ 7.53–7.47 (m, 1H), 7.45–7.36 (m, 2H), 7.34–7.28 (m, 1H), 7.28–7.19 (m, 2H), 7.10–7.01 (m, 2H), 3.26 (t, *J* = 6.7 Hz, 2H), 2.74 (t, *J* = 6.7 Hz, 2H), 2.61–2.51 (m, 2H), 2.31–2.18 (m, 2H), 1.72–1.60 (m, 2H); MS (APCI) *m/z* 366.1 [M + H]^+^.

#### N-(3,5-dibromo-4-hydroxyphenethyl)-1-(4-fluorophenyl)cyclobutane-1-carboxamide (PCB-31)

Method B (56%): ^1^H NMR (400 MHz, DMSO-*d*_6_) δ 7.29–7.19 (m, 4H), 7.12–7.00 (m, 2H), 3.17 (t, *J* = 6.5 Hz, 2H), 2.57 (ddd, *J* = 11.4, 8.2, 6.2 Hz, 4H), 2.31–2.20 (m, 2H), 1.78–1.62 (m, 2H), MS (APCI) *m/z* 472.0 [M + H]^+^.
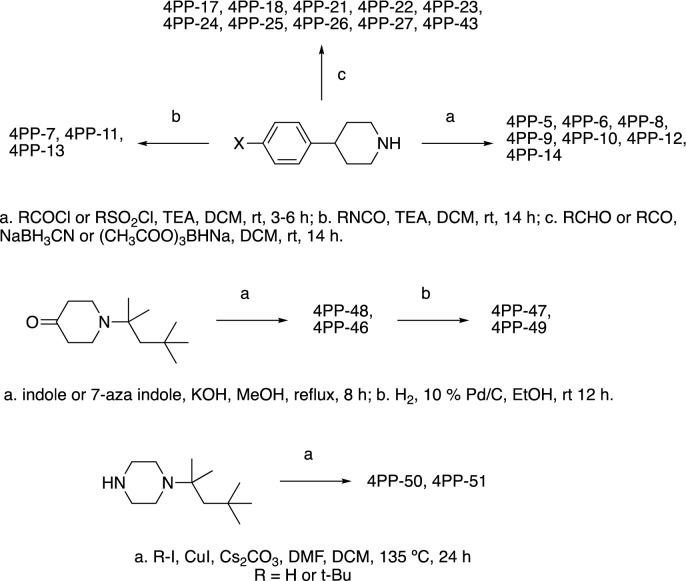


## Data Availability

Data supporting the results in the paper are located within the figures and tables. Data are reported as average and standard deviation; the raw data are available from the corresponding author on reasonable request.
